# Exposure of human cerebral microvascular endothelial cells hCMEC/D3 to laminar shear stress induces vascular protective responses

**DOI:** 10.1186/s12987-022-00344-w

**Published:** 2022-06-03

**Authors:** Nina Choublier, Meryam Taghi, Marie-Claude Menet, Morgane Le Gall, Johanna Bruce, Philippe Chafey, François Guillonneau, Amélie Moreau, Claire Denizot, Yannick Parmentier, Samir Nakib, Didier Borderie, Haniaa Bouzinba-Segard, Pierre-Olivier Couraud, Sandrine Bourdoulous, Xavier Declèves

**Affiliations:** 1 INSERM, Optimisation Thérapeutique en Neuropsychopharmacologie, Université Paris Cité, 75006 Paris, France; 2 Institut de Chimie Physique, CNRS, Université Paris-Saclay, 91405 Orsay, France; 3 3P5 Proteom’IC Facility, Institut Cochin, INSERM, CNRS, Université de Paris, F-75014 Paris, France; 4 Technologie Servier, F-45000 Orléans, France; 5 Service de Biochimie, Hôpital Cochin, Assistance Publique-Hôpitaux de Paris, 75014 Paris, France; 6 CNRS, INSERM, Institut Cochin, Inserm, CNRS, Université Paris Cité, 75014 Paris, France; 7 Biologie du Médicament Et Toxicologie, AP-HP, Hôpital Cochin, 75014 Paris, France

**Keywords:** Human endothelial cells, Proteomics, Shear stress, Blood–brain barrier, Brain microvessels

## Abstract

Endothelial cells (ECs) are constantly submitted in vivo to hemodynamical forces derived from the blood circulation, including shear stress (SS). ECs are able to detect SS and consequently adapt their phenotype, thus affecting many endothelial functions. If a plethora of shear stress-regulated molecular networks have been described in peripheral ECs, less is known about the molecular responses of microvascular brain ECs which constitute the blood–brain barrier (BBB). In this work, we investigated the response of human cerebral microvascular ECs to laminar physiological shear stress using the well characterized hCMEC/D3 cell line. Interestingly, we showed that hCMEC/D3 cells responded to shear stress by aligning perpendicularly to the flow direction, contrary to peripheral endothelial cells which aligned in the flow direction. Whole proteomic profiles were compared between hCMEC/D3 cells cultured either in static condition or under 5 or 10 dyn.cm^−2^ SS for 3 days. 3592 proteins were identified and expression levels were significantly affected for 3% of them upon both SS conditions. Pathway analyses were performed which revealed that most proteins overexpressed by SS refer to the antioxidant defense, probably mediated by activation of the NRF2 transcriptional factor. Regarding down-regulated proteins, most of them participate to the pro-inflammatory response, cell motility and proliferation. These findings confirm the induction of EC quiescence by laminar physiological SS and reveal a strong protective effect of SS on hCMEC/D3 cells, suggesting a similar effect on the BBB. Our results also showed that SS did not significantly increase expression levels nor did it affect the localization of junctional proteins and did not afect either the functional activity of several ABC transporters (P-glycoprotein and MRPs). This work provides new insights on the response of microvascular brain ECs to SS and on the importance of SS for optimizing in vitro BBB models.

**Supplementary Information:**

The online version contains supplementary material available at 10.1186/s12987-022-00344-w.

## Introduction

Endothelial cells (ECs) lining the blood vessels are able to physiologically adapt to variations of their environment from both the blood and surrounding tissues. The blood flow circulation is responsible for hemodynamic forces exerted on the endothelial wall. Among these forces, shear stress (SS) is a mechanical force tangentially applied on the endothelial cell surface. ECs detect and respond to this mechanical force by eliciting various downstream biochemical signals. This mechano-transduction process leads to the induction of specific pathways that control a plethora of cellular properties under both physiological and pathophysiological conditions [1, 2]. Indeed, endothelial responses are highly dependent upon the blood flow characteristics [3–5]. In physiological conditions, SS is laminar, with values varying from 5 to 30 dyn.cm^−2^ along the vascular tree [3]. At arterial curvatures and branching, where the blood flow is disturbed, ECs exhibit high rates of proliferation and a cobblestone morphology. Disturbed SS is associated with endothelial dysfunctions and pathogenesis processes in the cardiovascular system, such as atherosclerosis, thrombosis and aneurysm [6, 7]. Efforts were then made to implement physiological SS in EC cultures in vitro by using fluidic devices. Transcriptomic and proteomic studies, as well as functional assays confirmed that SS modulates various endothelial functions [2, 4, 8, 9] However, comparisons between the different studies were challenged by the heterogeneity of the nature, magnitude, and duration of SS exposure, but also by the cell sources and types. Along the vascular tree, ECs differently express some proteins and surface markers and are exposed to various magnitudes of SS. Whereas the mechano-transduction phenomenon was mostly investigated in the context of atherosclerosis, we still do not know if arteries, veins, and capillaries share the same endothelial responses to flow. To date, only about 50% of genes sensitive to SS have been reported to be shared by human ECs originating from umbilical vein and coronary artery [10]].

The blood–brain barrier (BBB) is primarily composed of brain microvascular endothelial cells (BMEC), which constitute the main interface between the blood and the central nervous system and display a unique phenotype compared with peripheral EC, including physical and metabolic characteristics [11, 12]. In the brain microvasculature, SS is considered laminar with a value of about 5 to 23 dyn.cm^−2^ [13–15]. The response to SS has been poorly studied in BMEC, and experimental fluidic studies mainly focused on the changes in barrier properties [16–18]. Immortalized human cerebral microvascular endothelial cells hCMEC/D3 are the most widely used and characterized human BMEC cell line, by us and others. They exhibit major characteristics of the BBB, such as expression of junctional proteins, uptake and efflux transporters, and other BMEC markers [19]. In the current work, we investigated the impact of SS on some key BBB characteristics of hCMEC/D3 cells, including cell morphology and orientation, expression of junctional proteins and ATP-binding cassette (ABC) efflux transporters. Furthermore, since whole proteomic analysis of the human BMEC responses to SS has not yet been reported, here we compared the whole proteome of hCMEC/D3 cell line cultured in static or SS conditions and used proteomic tools to evidence the main biological functions and pathways affected by SS.

## Materials and methods

### Materials

Invitrogen, Corning, and Gibco products were purchased from ThermoFisher scientific (Illkirch, France).

### Cell culture

The immortalized human cerebral microvascular endothelial cell line hCMEC/D3 was grown in Corning flasks after coating with 150 µg mL^−1^ rat collagen type I (R&D Systems, Bio-techne, Lille, France) in cold water (ThermoFisher scientific). Culture medium consisted of Endothelial Basal Medium-2 (Lonza, Basel, Switzerland) supplemented with 5% fetal bovine serum (Eurobio-Ingen, Les Ulis, France), 1% penicillin–streptomycin (Gibco), 1% chemically defined lipid concentrate (Gibco), 10 mM HEPES buffer (ThermoFisher scientific), 5 µg mL^−1^ ascorbic acid (Sigma-Aldrich, Saint Quentin Fallavier, France), and 1.4 µM hydrocortisone (Sigma-Aldrich). 1 ng mL^−1^ basic fibroblast growth factor (Sigma-Aldrich) was added extemporaneously.

### Fluidic experiments

hCMEC/D3 cells at passage between 24 and 26 were seeded at a density of 60 000 cells per cm^2^ on Ibidi collagen IV-coated microslides with 0.4 mm height (Ibidi, Munchen, Germany). After cells reached confluence, medium was supplemented by lithium chloride at 10 mM and resveratrol at 10 µM (Sigma-Aldrich) and microslides for the dynamic condition were connected to the Ibidi pump system. A shear stress of 5 or 10 dyn.cm^−2^ was applied for 72 h with recirculating culture medium. For static control condition, microslides were incubated in Olaf humidifying chambers (Ibidi) and culture medium was renewed every 24 h.

### Whole proteomic analysis

#### Sample preparation

After 72 h, cells were lysed with a solution containing 0.1% SDS (sodium dodecyl sulfate) in Tris–HCl 1.5 M pH 8.8 and snap frozen. One replicate consists of the pool of two microslides to reach 0.4 M cells (roughly 60 µg proteins). Four or five replicates were used per condition for the proteomic analysis. Bottom-up experiments’ tryptic peptides were obtained by Strap Micro Spin Column according to the manufacturer’s protocol (Protifi, NY, USA). Briefly: 30 μg of proteins from the above lysate were diluted in a single step reducing and alkylating reaction (400 mM TEAB (tetraethylammonium bromide), pH 8.5, 4% SDS, 20 mM TCEP (tris(2-carboxyethyl)phosphine), 100 mM chloroacetamide) and heated for 5 min at 95 °C. Denatured proteins were digested during 14 h at 37 °C with 1 µg Trypsin sequencing grade (Promega). After speed-vaccum drying of eluted peptides, these were solubilized in 2% trifluoroacetic acid (TFA) and fractionated by strong cationic exchange (SCX) StageTips, mainly as described [20].

#### Liquid chromatography-coupled mass spectrometry analysis (LC–MS)

LC–MS analyses were performed on a Dionex U3000 RSLC nano-LC- system (ThermoFisher scientific) coupled to a Q-Exactive mass spectrometer (ThermoFisher scientific). After drying, peptides from SCX StageTip, the five fractions were solubilized in 10 μL of 0.1% TFA containing 2% acetonitrile. One μL was loaded, concentrated and washed for 3 min on a C18 reverse phase precolumn (3 μm particle size, 100 Å pore size, 75 μm inner diameter, 2 cm length, from ThermoFisher Scientific). Peptides were separated on an Aurora C18 reverse phase resin (1.6 μm particle size, 100 Å pore size, 75 μm inner diameter, 25 cm length (IonOpticks, Middle Camberwell Australia) with a 170 min run-time gradient ranging from 99% of solvent A containing 0.1% formic acid in milliQ-grade H_2_O to 40% of solvent B containing 80% acetonitrile, 0.085% formic acid in mQH2O. The mass spectrometer acquired data throughout the elution process and operated in a data-dependent scheme with full MS scans acquired, followed by up to 10 successive MS/MS HCD-fragmentations on the most abundant ions detected. Settings for Q-Exactive were: full MS AGC target 1.10^6^ with 60 ms maximum ion injection time (MIIT) and resolution of 70 000. The MS scans spanned from 350 to 1500 Th. Precursor selection window was set at 2 Th. HCD Normalized Collision Energy (NCE) was set at 27% and MS/MS scan resolution was set at 17,500 with AGC target 1.10^5^ within 60 ms MIIT. Dynamic exclusion time was set to 30 s and spectra were recorded in profile mode.

#### Protein quantification and comparison

The mass spectrometry data were analyzed using Maxquant version 1.6.17 [21]. The database used was a concatenation of Homo sapiens sequences from the Swissprot databases (release 2020-10) and an in-house list of frequently found contaminant protein sequences. The precursor and fragment mass tolerance were set to 20 ppm. Carbamidomethylation of cysteins was set as permanent modification and acetylation of protein N-terminus and oxidation of methionines were set as variable modification. Second peptide search was allowed and minimal length of peptides was set at seven amino acids. False discovery rate (FDR) was kept below 1% on both peptides and proteins. Label-free protein quantification (LFQ) was done using both unique and razor peptides. At least 2 such peptides were required for LFQ. The “match between runs” (MBR) option was allowed with a match time 0.7 min window and an alignment time window of 20 min. For differential analysis, LFQ results from MaxQuant were quality-checked using PTXQC [22], imported into the Perseus software (version 1.6.14) [23] Reverse and contaminant proteins were excluded from analysis. A protein was considered as significantly differentially expressed between two conditions when it was quantified in only one condition (ON and OFF proteins), or when: (1) the fold change is superior or inferior to 1.2, (2) ANOVA p-value is inferior to 0.01, and (3) Student t-test p-value is inferior to 0.01.

Venn diagram were designed using FunRich (http://www.funrich.org/), and graphs were made with GraphPad Prism.

#### Ingenuity pathway analyses

Data were first analyzed through the use of Ingenuity Pathway Analysis (IPA, QIAGEN Inc., https://www.qiagenbioinformatics.com/products/ingenuitypathway-analysis) version 60,467,501. Significantly overrepresented terms (canonical pathways, functions, upstream regulators) were identified with a right-tailed Fisher’s Exact Test that calculates an overlap p‐value determining the probability that each term associated with our lists of differential proteins was due to chance alone. The z-score is a statistical measure of correlation between relationship direction (activation or inhibition) and experimental protein expression. Its calculation assessed the activation (positive z-score) or repression (negative one) of each term. To be considered significant the z-score has to be greater than 2 in absolute value. Enrichments were further explored using Gene Set Enrichment Analysis (GSEA) 4.1.0 software (https://www.gsea-msigdb.org/gsea/) [24, 25]. GSEA determined whether gene sets from the Molecular Signatures Database (MSigDB) Wikipathways collection (v7.4) were randomly distributed throughout our ranked protein lists or if they were located at the top or bottom of them. For these pathway analyses, selection criteria were enlarged (with p-values until 0.05) to provide an overview of the pathways and functions affected by SS.

### Quantification of BBB proteins by targeted absolute quantitative proteomics by LC–MS/MS

Targeted absolute proteomic analysis was performed as previously described for hCMEC/D3 cells and human BBB materials [26, 27] to compare protein levels of the P-glycoprotein (P-gp), multidrug resistance-associated proteins 1 and 4 (MRP1 and MRP4), Claudin-5, and PECAM-1 proteins in hCMEC/D3 cultured in absence or presence of SS. Cells were rinsed twice in DPBS, extracted from microslides with trypsin at 0.25%, and centrifuged 10 min at 10 000 g 4 °C to freeze the cell pellet. After resuspension in denaturing buffer (7 M guanidine hydrochloride, 10 mM EDTA, 500 mM Tris pH 8.5), proteins were extracted by sonication cycles of 5 min using a BioRuptor (Diagenode, Belgium) at high frequency, and centrifuged at 10,000 g at 4 °C for 10 min to collect supernatants (post-nuclear fraction). Then, proteins were reduced by dithiothreitol and alkylated by iodoacetamide. The protein samples were subsequently precipitated in a mixture of methanol and chloroform, and resolubilized with 6 M urea in 0.1 M Tris–HCl pH 8.5. The pellets were resuspended in 6 mol.L^−1^ urea and 0.2% (W/V) ProteaseMaxTM detergent. After a 10 min incubation and agitation at room temperature, the samples were diluted with 0.1 mol.L^−1^ Tris buffer (pH 8.5) to a final urea concentration of 1.4 mol.L^−1^ and 0.05% (W/V) of ProteaseMaxTM before sonicating for a complete resuspension. rLysC endoprotease was added to the samples in an enzyme-protein mass ratio of 1:50 and digested at room temperature for 3 h. A second protein digestion using trypsin solution was performed at 37 °C overnight. Peptides were synthetized in light and heavy forms as standards by Pepscan (Lelystad The Netherlands). A calibration curve was prepared in a mix containing 10% acetonitrile and 0.1% formic acid in water, including the light peptides covering a range from 0.125 to 150 fmol.µL^−1^ and the heavy peptides in constant concentration (100 fmol µL^−1^). Heavy peptides were also added in samples after digestion for absolute quantification. Samples were dried using a centrifugal vacuum concentrator (Maxi-Dry Lyo, Heto Lab Equipment, Roskilde, Denmark), stored at – 80 °C and solubilized just before analysis in an aqueous mixture containing 10% acetonitrile plus 0.1% formic acid. Targeted liquid chromatography with tandem mass spectrometry (LC–MS/MS) analyses were performed employing an ACQUITY UPLC H-Class® System in line with a Waters Xevo^®^ TQ-S mass spectrometer (Waters, Manchester, UK) operated in multiple reaction monitoring (MRM) mode. Skyline software (version 20.1.0.155) was used to export the area ratios of light to labeled peptides. Protein expression levels were based to calibration curves and normalized with expression of the Na^+^/K^+^-ATPase membrane marker [28]. The detail of each proteotypic peptide is provided in Additional file 2: Table S1.

### Immunostaining

Immunofluorescence staining of NRF2, *adherens* (PECAM-1, VE-Cadherin, β- and γ-catenins) and tight (ZO-1, Occludin, Claudins-5 and -11) junction proteins was performed in microslides. After washings with DPBS, cells were fixed with 3.2% paraformaldehyde (Electron Microscopy Sciences, Hatfield, PA, USA) for 10 min, and permeabilized with 0.1% Triton X-100 (ThermoFisher scientific) for 10 min. Primary antibody references are given in Additional file 2: Table S2. Primary antibodies were diluted at 1:200 in 2% Bovine Serum Albumin (BSA) and incubated for 1 h. A mix of Alexa Fluor 488 Phalloidin (labelling F-actin) (Invitrogen) at 1:500, anti-rabbit IgG Alexa Fluor 633 and anti-mouse IgG Alexa Fluor 555 secondary antibodies (Invitrogen) at 1:500, and 1 µg/mL hoechst 33,342 (labelling nuclei) (Invitrogen) with 2% BSA was incubated for 1 h in the dark. Between all incubations, channels were washed three times with DPBS. Microslides were filled with Ibidi Mounting Medium (Ibidi) and preserved at 4 °C until acquisition.

#### Image acquisition

Immunostaining was analyzed by confocal imaging. The spinning disk system Yokogawa CSU-W1 integrated in Metamorph software was mounted to the camera port Nikon Ti-E inverted microscope (20 × Plan Fluor NA 0.45 and 60 × Plan Apo NA 1.27 water objective lenses). Images were acquired by a 16-bit cooled sCMOS camera (Prime 95B, Photometrics). For each condition, a Z-dimension series of images were acquired from four random locations. All images were taken with the same acquisition parameters and processed using ImageJ software (NIH). No detectable signal was observed when primary antibodies were omitted.

#### Orientation analysis

We worked on greyscale confocal images of actin fibers and cell membranes, acquired after phalloidin and β-catenin staining respectively. The alignment was corrected by rotation of 2.5 degrees so that the sidewalls of the channel precisely correspond to the verticality (represented by 90°). We used the OrientationJ set of plug-ins available on ImageJ Software. OrientationJ Analysis created color-coded overlays which pixel hue corresponded to the angle of local staining orientation. OrientationJ Distribution computed in a spreadsheet the numeric orientation values ranged from 90°  to 90°  relative to the horizontal. We converted this range from 0 to 180°  to match the verticality (corresponding to the channel axis/flow direction) with 0° . The number of structures in each direction was next plotted as angle histogram using the rose function in Matlab. We represented the total number of pixels of F-actin structures according to their orientation in a polarplot.

### Efflux transporter activity

P-gp and MRP transport activities were determined by quantifying intracellular accumulation of their substrates in the absence of presence of specific inhibitors. Non-fluorescent lipophilic molecules calcein-acetoxymethyl (calcein-AM) and 5(6)-carboxy-2ʹ,7ʹ-dichlorofluorescein diacetate (DCFDA) (Sigma-Aldrich) were used to monitor P-gp and MRP efflux activities, respectively [29]. Calcein-AM, a P-gp substrate, is intracellularly cleaved by endogenous esterases in calcein, a hydrophilic fluorescent metabolite that accumulates within the cytosol [30]. DCFDA is metabolized in carboxyfluorescein, a fluorescent substrate of MRPs transporters [31]. Valspodar (PSC833, Sigma-Aldrich) and MK-571 (Sigma-Aldrich) were used as potent inhibitors of P-gp and MRPs transporters, respectively [29, 32]. All the incubation solutions were diluted in HBSS buffer with 1% HEPES with the same amount of DMSO (0.3% v/v max). Cell monolayers in microslides were disconnected from the perfusion and experiments were initiated by washing the cells with DPBS and incubating them with 10 µM Valspodar or 100 µM MK-571 or vehicle (DMSO) for 10 min. Then, respective substrates (1 µM calcein-AM or 3 µM DCFDA) were added in the incubation buffer. After incubation 30 min at 37 °C, reaction was stopped by addition of ice-cold DPBS with two washings. Cells were then extracted from microslides with a solution containing 0.1% SDS in Tris 1.5 M pH 8.8. Then, cell lysates were transferred in a 96-well plate to quantify intracellular substrate accumulation by fluorescence through a 485 nm band pass filter (Victor, Perkin Elmer). Protein concentrations were determined for each replicate using BCA Protein Assay kit (ThermoFisher scientific). Normalization of fluorescence intensities to the quantities of total proteins avoided potential variation in cell number. Three experiments including three replicates were independently performed for each condition. The difference in cell accumulation of P-gp or MRP substrates with and without specific inhibitor reveals the activity of each transporter in the substrate efflux. GraphPad Prism software was used for statistical analysis and graphical representation. Numerical results are given as means ± standard deviation. Data were treated using two-way ANOVA and post-hoc Tukey's test. Statistical significance was accepted for *p < 0.05; **p < 0.01; ***p < 0.001.

### Measurement of intracellular glutathione (GSH) by LC–MS/MS

Determination of intracellular GSH was performed using liquid chromatography with tandem mass spectrometry (LC–MS/MS) adapted from [33]. GSH and N-ethylmaleimide (NEM) were purchased from Sigma Aldrich. Solvents for sample preparation and LC–MS/MS analysis were HPLC grade. Briefly, NEM was in vitro conjugated to GSH (GS-NEM) and GS-NEM was further used for the LC–MS/MS calibration curve. hCMEC/D3 cells were seeded at a density of 60,000 cells per cm^2^ on Ibidi collagen IV-coated microslides as described above and after reaching confluence, they were exposed to a shear stress of 10 dyn.cm-^2^ for 72 h. Cells were then washed two times with PBS containing 1 mM NEM, then lysed with methanol 80% (v/v) for 10 min to release intracellular GS-NEM contents. Cell extracts were stored at – 80 °C until LC–MS/MS analysis. Stable isotope-labeled (phenylalanine-^13^C6) was acquired from Sigma Aldrich. MS/MS quantification was performed using a TSQ Quantis Triple Quadrupole Mass Spectrometer (ThermoFisher). LC separation was achieved on a HSS T3 column (100 × 2.1 mm, 2.5 µm, Waters) using a mobile phase composed of water containing ammonium formiate 2 mM at pH 3.1 (A) and acetonitrile containing 0.1% formic acid (B) in gradient elution mode at a flow rate of 0.25 mL/min. Quantification of NEM-GS in cell extracts was achieved with a calibration curve of GS-NEM (25 to 500 mM) with positive mode and MRM transitions (m/z) of 433.2/303.9.

## Results and discussion

### Shear stress induced hCMEC/D3 alignment perpendicular to the flow direction

**Fig. 1:**
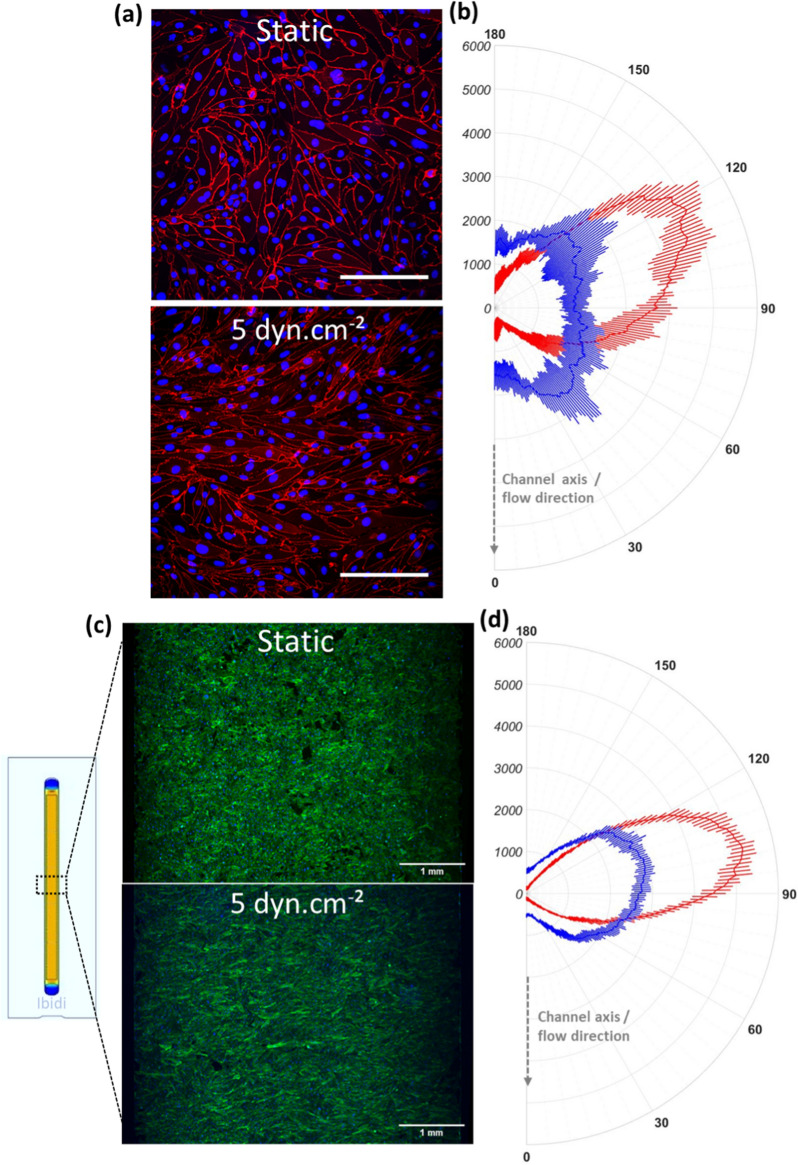
Cellular orientation of hCMEC/D3 cells cultured either in static or dynamic (5 dyn.cm^−2^ shear stress) condition. This figure shows β-catenin staining (scale = 200 µm) (**a**) and F-actin staining throughout the channel width (scale = 1 mm) (**c**), and their respective orientation analysis (**b**) and (**d**). Cells were seeded in Ibidi microslides and cultured either in static condition or under a shear stress of 5 dyn.cm^−2^ for 72 h. Staining was performed with an antibody against the cell membrane protein -catenin, phalloidin targeting filamentous actin (F-actin). Nuclei were stained in blue with Hoechst 33,342 in order to compare images with a similar confluence. Images were acquired by confocal microscopy, and each image is representative of 4 images acquired in random locations. Four images per condition were analyzed with OrientationJ in ImageJ software. This plug-in gives the orientation of each pixel from 0° to 180° relative to the channel axis (i.e. the flow direction). 0° and 180° correspond to the channel/flow direction, whereas 90° is the perpendicular to the flow. The respective angles of distribution are graphed in polarplot as means $\pm$ standard deviation. Blue: static condition, red: shear stress condition (5 dyn.cm^−2^ exposure)

hCMEC/D3 cells recapitulate many features of the endothelial cells from the BBB, including their morphology in a spindle shape [19]. The impact of SS exposure on hCMEC/D3 cell morphology was first analyzed. Cells were cultured in Ibidi microslides under static condition until confluence, then either in static or fluidic condition at 5 dyn.cm^−2^ for 72 h. Observation by phase contrast microscopy revealed a more uniform alignment of cells as early as a few hours after SS exposure that was maintained for 72 h. In case of return to static culture, after stopping the flow, cells readopt their initial phenotype (with a more random orientation) within a few hours. These observations suggest that SS has a reversible effect on cytoskeleton change. Images in Fig. 1a confirmed our previous observation because cells appeared more elongated (fusiform) under flow, as compared to cells that remained in static condition. Based on these images, we performed an orientation analysis with ImageJ software in order to quantify the directionality of the cells. Angular distributions were computed from 0° to 180°, with 0° and 180° being the channel axis (corresponding to the flow direction), and 90° corresponds to the perpendicular of the channel/flow axis. Our results in Fig. 1b showed that cells under static condition had no preferential orientation. In contrast, cells exposed to SS exhibited a significant preferential alignment from 100° to 130° relative to the flow axis. Then, the effect of SS on the actin cytoskeleton was investigated. In static culture, F-actin fibers visually appeared randomly oriented at any time, without homogeneous orientation (Fig. 1c**)**. When submitted to SS, cells had a more pronounced orientation between 60° and 130°, with significantly less fibers aligned with the flow (0°) (Fig. 1d**)**. For both static and fluidic conditions, the peak of actin orientation was located around 105°. In SS condition at 5 dyn.cm^−2^, this phenomenon was even more marked, with almost a two-fold increase in the number of fibers oriented between 90° and 120°. The differences in orientation between static and SS conditions were statistically significant (Student p-values < 0.05) and observed at both 5 and 10 dyn.cm^−2^ (Additional file 2: Figure S1). In conclusion, analysis of both cellular and F-actin orientations confirmed the SS influence on cytoskeleton and endothelial cell organizations.

Cellular and cytoskeletal alignments are responses previously reported in vitro with peripheral endothelial cells cultured under flow [34–38]. These studies demonstrated that shear force reduced the endothelial cell movement. Actin fibers aligned with the flow and cells reoriented in the same direction. This F-actin reorganization might be responsible for the decrease of compliance of the endothelial cell membrane [39]. Cell alignment with the flow corresponds to those observed in vivo in dog, rat, and rabbit aortic endothelial cells [40–44]. Regarding the morphological response of BMEC to SS however, there is no consensus [45, 46]. Several studies reported that BMEC do not operate any morphological change upon SS [45, 47, 48], Searson’s team showed that human BMEC and iPSC-derived human BMEC resist elongation due to SS [37, 49, 50], whereas one study highlighted a perpendicular orientation to the flow in response to SS exposure [49]. In accordance with this work, we observed here a perpendicular orientation of the hCMEC/D3 cells under SS. A possible explanation of this observation is that brain capillary endothelial cells could wrap around themselves in the radial direction to limit the length of tight junctions [49]. This fact is consistent with the in situ observation of endothelial cell organization at the BBB level, one cell closing the microvascular lumen by forming junctions with itself [12, 51], which might correlate with the formation of TJs and the reduction of the paracellular permeability.

### Shear stress induced significant changes in protein expression of hCMEC/D3 cells

**Fig. 2:**
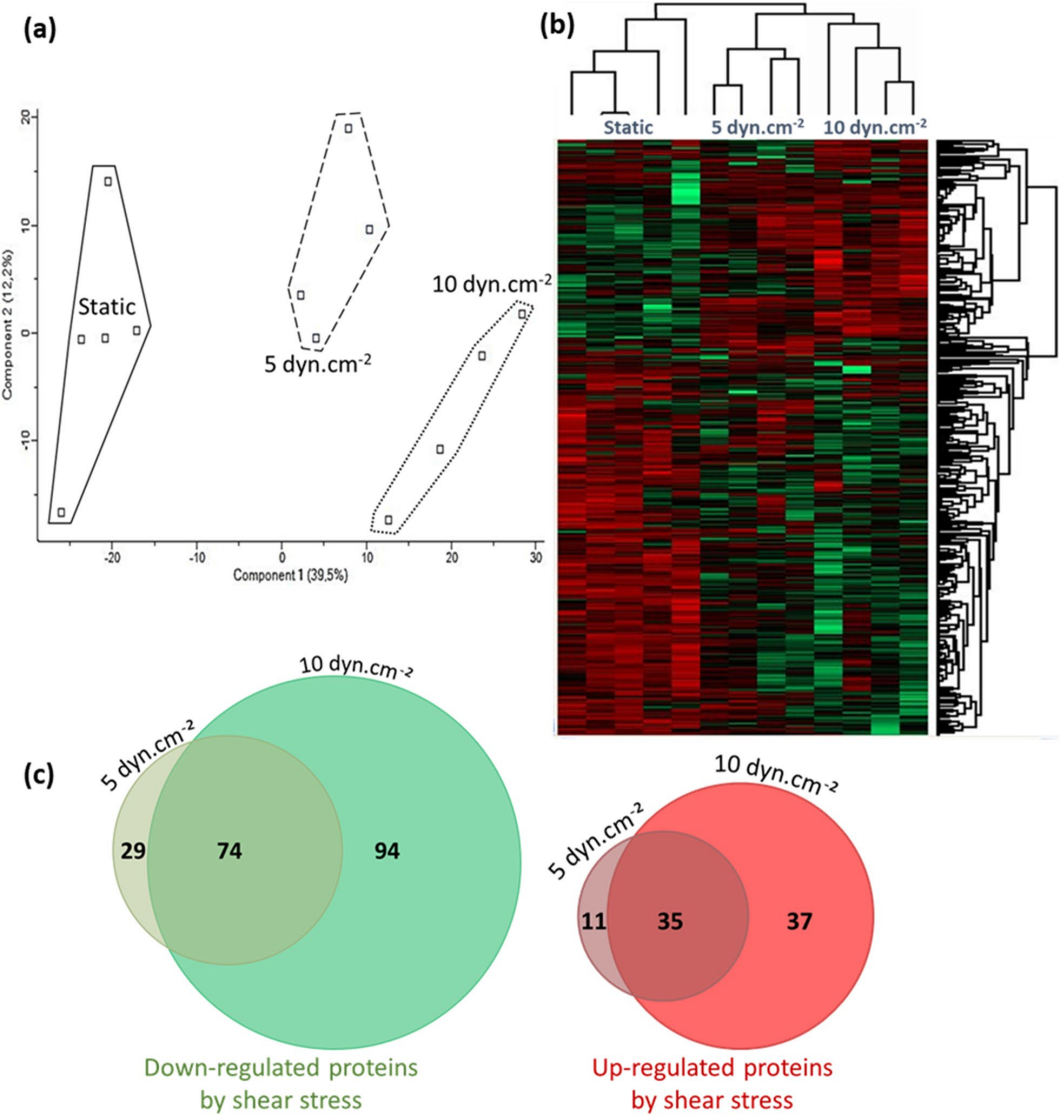
Label free whole proteomics from hCMEC/D3 cells cultured in static condition or under shear stress of 5 or 10 dyn.cm^−2^. Chosen criteria were |fold change|> 1.2 and both ANOVA and Student T-test p-values < 0.01 plus ON/OFF proteins. **a** Principal component analysis (**b**) Heatmap of significantly changed proteins between culture conditions. Red and green highlight respectively increased and decreased proteins. **c** Venn diagrams of differentially expressed proteins at 5 and 10 dyn.cm^−2^ compared to the static culture condition

hCMEC/D3 cells were cultured either in a static condition or under a laminar flow for 72 h at 5 or 10 dyn.cm^−2^. To evaluate the impact of SS exposure on the protein expression profiles, the proteomes of the three types of cultures were analysed. A total of 3592 proteins was identified. The principal component analysis (Fig. 2a) distinguishes each condition by using the highest statistically significant proteins. It highlights that SS exposure leads to significant variations in the proteomic profile of hCMEC/D3 cells. Moreover, these changes appear to be dependent on the SS magnitude, as the protein expression profile at 10 dyn.cm^−2^ is even more distant from the static profile than the one at 5 dyn.cm^−2^. Heatmap in Fig. 2b shows that a higher number of proteins are down-regulated by the SS compared with up-regulated proteins.

The Venn diagram (Fig. 2c) confirmed that SS at 10 dyn.cm^−2^ exhibited the highest number of differentially expressed proteins. 74 proteins were significantly downregulated in both SS conditions compared to the static culture, including 7 proteins no longer detected at 5 and 10 dyn.cm^−2^. Reciprocally, 35 proteins were significantly up-regulated by SS exposure, two of them being detected only in SS conditions. These proteins were identified and listed in Additional file 2: Table S3. We can note that 4.1% and 6.7% of the static proteome was significantly affected after SS exposure at 5 and 10 dyn.cm^−2^, respectively. Moreover, 3.0% of the static proteome significantly changed in a common way in both fluidic conditions.

**Fig. 3:**
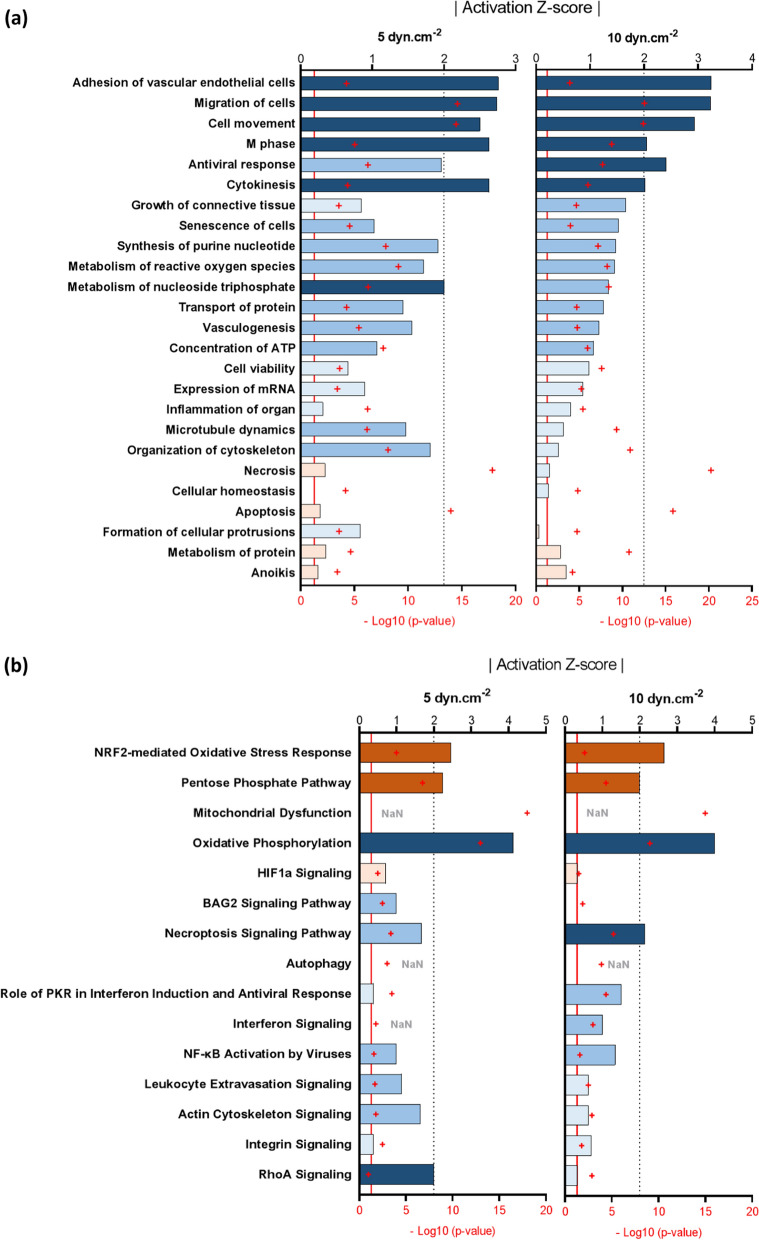
Principal significant enriched (**a**) biological functions and (**b**) canonical pathways determined by Ingenuity Pathway Analysis. Left graphs give comparisons between proteomic profiling of hCMEC/D3 cells culture under 5 dyn.cm^−2^ shear stress compared to the static culture, and right graphs show comparisons between proteomic profiling of hCMEC/D3 cells exposed at 10 dyn.cm^−2^ shear stress compared to the static condition. Activation z-score depicts the predicted degree of activation (positive z-score in orange) or inhibition (negative z-score in blue). An enrichment is considered significant when − log_10_(p-value) > 1.3 or |activation z-score|> 2. *NaN* no activity pattern available, despite significant association of the proteins within the pathway

The proteomes of hCMEC/D3 cells under static or SS conditions were analysed using Ingenuity Pathway Analysis (IPA) to highlight overrepresented functions and canonical pathways (see following paragraphs). These pathways were obtained from identities of hCMEC/D3 proteins up- and down-regulated at 5 or 10 dyn.cm^−2^ with a p-value < 0.05 (EXCEL file, Additional file 2: Table S4). We focused our analysis on the common responses at 5 and 10 dyn.cm^−2^ versus static condition in order to describe the major pathways affected by SS. Most biological functions significantly affected by SS were inhibited (Fig. 3a). They include cell senescence, leukocyte transmigration, antiviral response, biosynthesis of nucleotide triphosphate, cell movement, cytokinesis, mitosis, and adhesion. Among the significant canonical pathways reported in Fig. 3b, NRF2-mediated oxidative stress response and pentose phosphate pathway were activated, whereas oxidative phosphorylation was predicted to be inhibited in both comparisons. Additional file 2: Table S5 provides a list of principal upstream regulators significantly identified by IPA.

### Shear stress strongly promoted an oxidative stress response

#### Activation of NRF2-mediated oxidative stress response

**Fig. 4:**
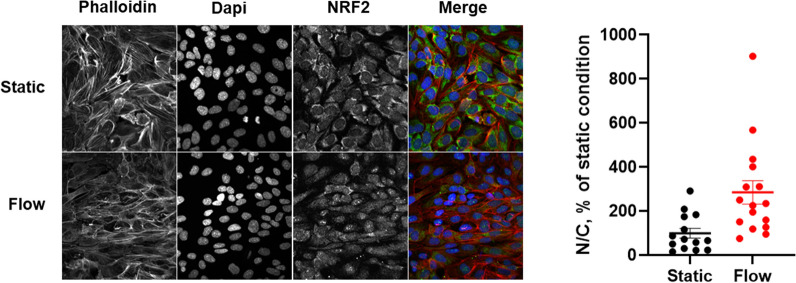
Impact of shear stress exposure on NRF2 protein localization. Left: cells were seeded in Ibidi microslides and cultured either in static condition or under a shear stress of 10 dyn.cm^−2^ for 72 h. Staining was performed with an antibody against NRF2, phalloidin and dapi. Shown are representive images of 14 images taken in random locations. Right: quantification of the mean fluorescence intensity of NRF2 in the nucleus and the cytosol was performed using Image J analysis on 14 images per condition (~ 50 cells per image). Results are presented as Nuclear/Cytosol (N/C) ratio in percentage of the control (static) condition

**Fig. 5:**
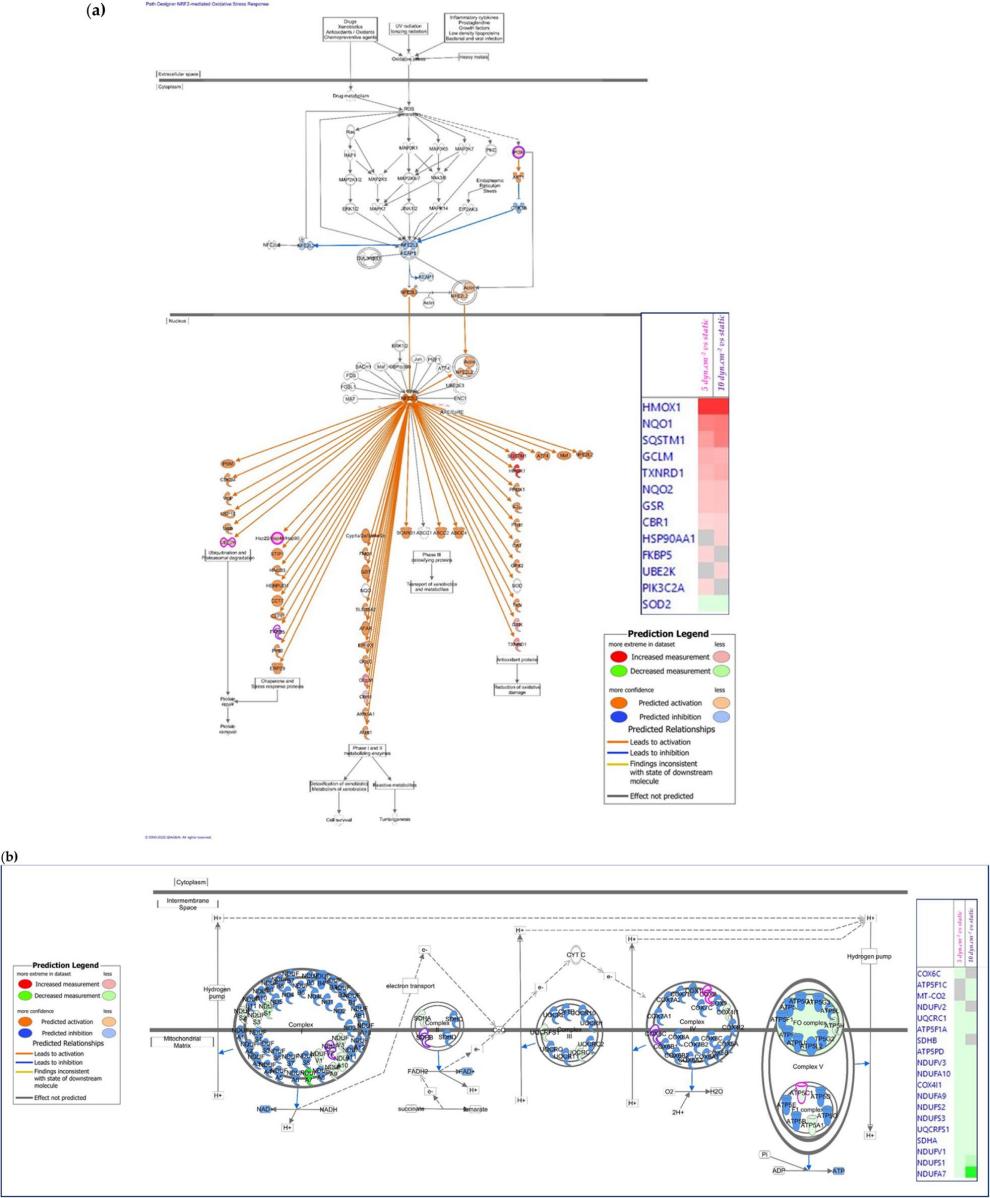
Schematic overview of some of the most significantly canonical pathways predicted by Ingenuity Pathway Analysis (Qiagen) to be affected by shear stress in hCMEC/D3 cells. **a** NRF2-mediated oxidative stress response and (**b**) Oxidative phosphorylation pathway. Analysis gives the common responses to shear stress exposure at both 5 and 10 dyn.cm^−2^ compared to the static condition. Relative changes in protein levels are depicted by graduated shades of color coding: red (up), green (down), white (no change or not applicable). Predictions are shown in orange (activation predicted) and blue (inhibition predicted). Proteins in surrounded of purple are modified only at 10 dyn.cm^−2^ vs static; and those surrounded of pink are modified only at 5 dyn.cm^−2^ vs static

IPA revealed that the most activated pathway in response to flow was “NRF2 (nuclear factor erythroid 2-related factor 2) -mediated oxidative stress response”, with an activation z-score > 2 in the 5 and 10 dyn.cm^−2^ SS conditions. Similarly, Gene Set Enrichment Analysis (GSEA) confirmed that “NRF2 pathway”, “nuclear receptor metapathway”, and “NRF2 regulation” were the most activated pathways in both 5 and 10 dyn.cm^−2^ conditions. NRF2 is a transcriptional factor that remains inactive in the cytosol at the steady state. It is activated in case of oxidative stress and exerts antioxidant and cytoprotective effects to counterbalance the synthesis of reactive oxygen species (ROS). NRF2 binds to DNA promoter sequences containing antioxidant response elements (ARE) and initiates gene transcription [52, 53]. ARE-genes encode for proteins involved in redox homeostasis, nicotinamide adenine dinucleotide phosphate (NADPH) regeneration, and detoxication [54]. In hCMEC/D3 cells, although NRF2 protein was not detected by proteomic analysis in any condition, we observed by immunofluorescence analysis that NRF2 localization in cells grown under static conditions was mainly cytosolic, whereas upon cells exposure to SS (10 dyn.cm^−2^ for 72 h) NRF2 accumulated in the nuclei (Fig. 4). In addition, our results showed that most of the proteins significantly upregulated by SS are encoded by ARE-genes and/or belong to pathways known to be activated by NRF2, as presented in Fig. 5a. These results are consistent with previous data showing, in peripheral EC, that laminar physiological SS (in contrast to disturbed flow) generates activation and nuclear accumulation of NRF2 as well as its binding to target genes [52]. Activation of NRF2 under flow was shown to persist after 48 h of flow [53].

#### Activation of the pentose phosphate pathway (PPP) for NADPH regeneration

**Fig. 6:**
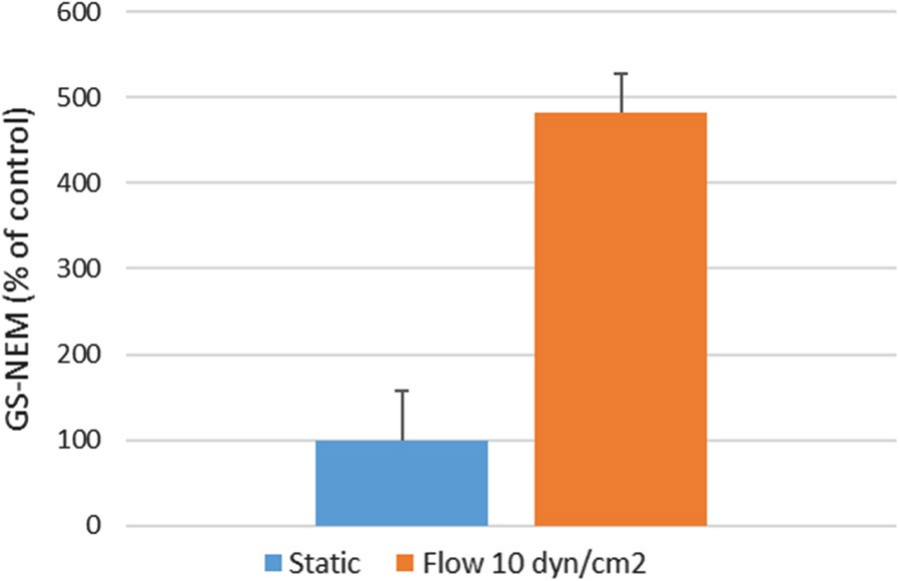
Impact of shear stress exposure on intracellular GSH contents in hCMEC/D3 cells. hCMEC/D3 cells were exposed for 72 h to a shear stress of 10 dyn.cm^−2^ and GSH cell content was further assessed using LC–MS/MS (see materials and methods). Histograms show GS-NEM concentrations as percentage (%) of control, with cells cultured under static condition. Three independent experiments were performed in triplicate. Statistical significance was determined by Student t-test (**p < 0.01)

NADP-dependent malic enzyme (ME1), glucose-6-phosphate dehydrogenase (G6PD), and 6-phosphogluconate dehydrogenase (PGD) are enzymes involved in the oxidative phase of pentose phosphate pathway (PPP) by generating NADPH, whereas transketolase (TKT) and transaldolase (TALDO1) catalyze the non-oxidative phase for the synthesis of nucleotides and glyceraldehyde-3-phosphate to restart the oxidative phase. All these proteins were upregulated by SS in our dataset (Additional file 2: Table S3). Moreover, PPP (both oxidative and non-oxidative phases) was predicted to be activated by SS according to IPA, with an activation z-score > 2. NRF2 was indeed described to positively regulate the PPP [55]. NADPH is used for antioxidant mechanisms. It is a source of electrons for reducing reactions, involving for example NAD(P)H dehydrogenase [quinone] 1 and 2 (NQO1 and NQO2) detoxification enzymes [56], thioredoxin reductase 1 (TXNRD1) in thioredoxin system [57], and glutathione reductase (GSR), glutamate–cysteine ligase regulatory subunit (GCLM), and SH3 domain-binding glutamic acid-rich-like protein 3 (SH3BGRL3) belonging to the glutathione-glutaredoxin (GST-Grx) system. All these proteins were significantly over-expressed by SS (Additional file 2: Table S3). GST-Grx system requires NADPH and glutathione (GSH) to generate antioxidant forms. Glutathione (GSH) is a neuroprotective antioxidant with a high reducing power [58], identified here as an enriched upstream regulator (activation z-score of 1,067 in both conditions, Additional file 2: Table S5). To confirm that hCMEC/D3 cells exposed to SS might be better protected from an oxidative stress, we quantified intracellular GSH in cells exposed or not to SS. Indeed, exposure of hCMEC/D3 cells to SS at 10 dyn.cm-^2^ for 72 h increased by 4.8-fold intracellular GS-NEM levels compared to control static cells (Fig. 6). This observation strongly suggests that NRF2-mediated upregulation of proteins by SS, as observed by proteomic analysis, may have functional consequences in terms of protection from oxidative stress. In summary, these observations indicate that SS induced activation of the PPP pathway in hCMEC/D3 cells, that might play a key role in NADPH regeneration.

#### Inhibition of the oxidative phosphorylation

Both IPA and GSEA revealed that the most inhibited pathway in response to flow was oxidative phosphorylation, as illustrated in Fig. 5b. Eight subunits of the complex I of the mitochondrial respiratory chain were significantly inhibited by SS (NADH dehydrogenases NDUFA9, -A10, -A11, -A12, -B4, -S1, -S2, and -V1, Additional file 2: Table S3b). These enzymes catalyze NADH oxidation into NAD^+^. Consequently, their inhibition is supposed to preserve NADPH for antioxidant process. Succinate dehydrogenase [ubiquinone] flavoprotein subunit A (SDHA), cytochrome c oxidase subunit 4 isoform 1 (COX4I1), and stomatin-like protein 2 (STOML2) also participate to the mitochondrial respiratory chain (respectively in complex I, IV, and by stimulating it [59]) and are significantly downregulated in both dynamic conditions (Additional file 2: Table S3b). These observations are in line with the inhibition of the respiratory chain, predicted by IPA. Mitochondrial dysfunction is a canonical pathway significantly enriched in both fluidic profiles compared to the static one (Fig. 3b). Mitochondrial respiratory chain is responsible for ROS and ATP synthesis mediated by ATP synthase subunit d (ATP5H), another protein significantly inhibited by SS (Additional file 2: Table S3b). The metabolism of ROS and concentration of ATP appeared significantly enriched functions with negative z-scores (Fig. 3a), suggesting a probable inhibition by SS.

All together, these results are consistent with the EC capacity to protect themselves from oxidative stress by multiple mechanisms. So far, only two publications reported the preservation of NADPH in response to SS in BMEC. RNA encoding NADH-producing enzymes were overexpressed whereas those encoding NADH-consuming enzymes were downregulated by SS in primary arterial ECs and BMEC [17, 60]. The present work is, to our knowledge, the first to demonstrate the implication of NRF2 in antioxidant pathways in BMEC under shear stress.

### Shear stress exerted a strong anti-inflammatory effect on hCMEC/D3 cells

**Fig. 7:**
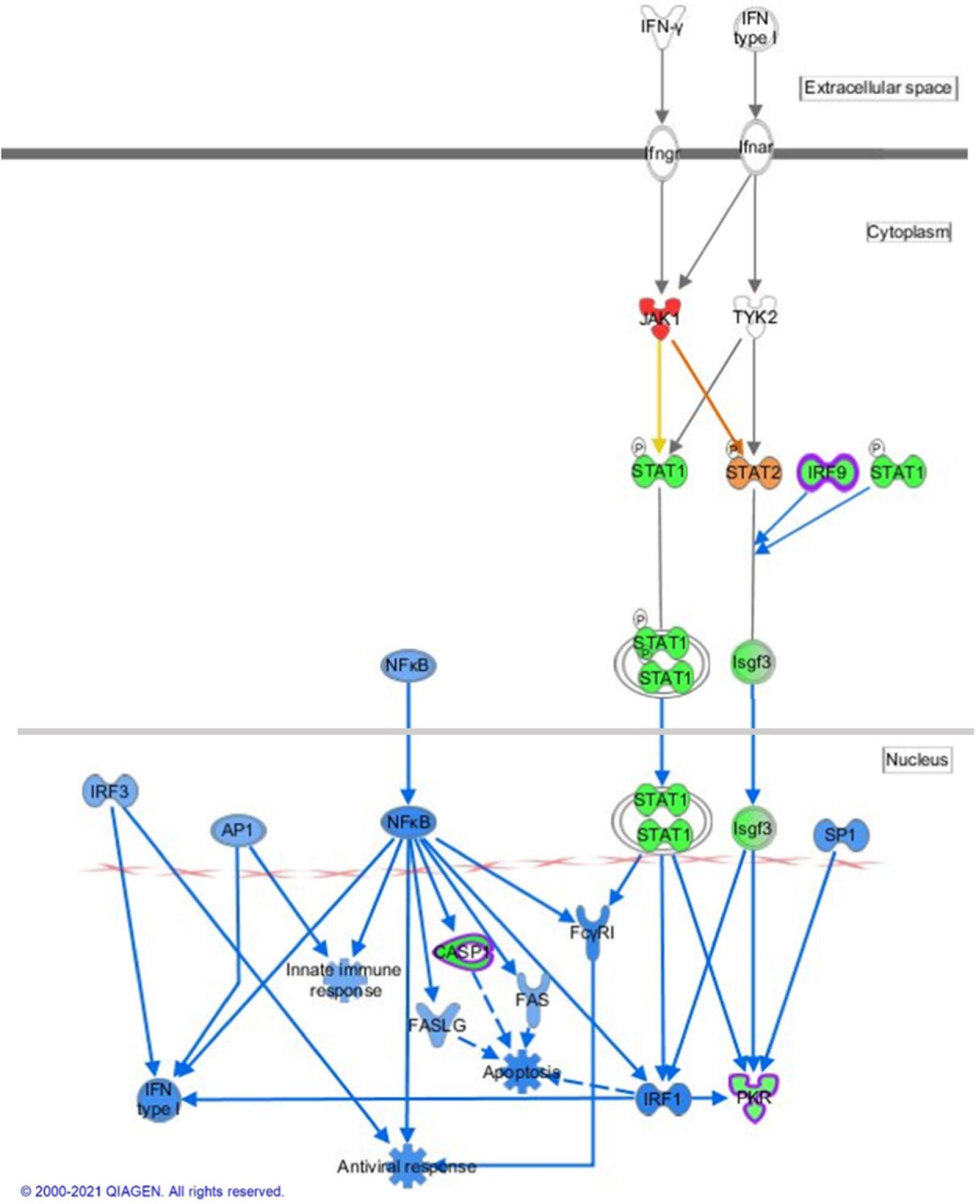
Schematic representation of the proteins involved in the interferon signaling induced in antiviral response that are significantly predicted by Ingenuity Pathway Analysis to be affected by the flow in hCMEC/D3 cells. Analysis gives the common responses to shear stress exposure at both 5 and 10 dyn.cm^−2^ compared to the static culture. Relative changes in protein levels are depicted by graduated shades of color coding: red, up; green, down; white, no change or not applicable; orange: activation predicted; blue: inhibition predicted. Proteins in surrounded of purple are modified only at 10 dyn.cm^−2^ vs static; and those surrounded of pink are modified only at 5 dyn.cm^−2^ vs static

IPA predicted the antiviral response as a function inhibited by SS, with activation z-scores of – 1.97 and – 2.41 at 5 and 10 dyn.cm^−2^, respectively. The biological function “inflammation of organ” is also diminished (z-scores –0 .31 and –0 .65) (Fig. 3a). Heme oxygenase-1 (HMOX1) was the most over-expressed protein regardless of the SS magnitude (fold changes of 10.4 and 16.3 at 5 and 10 dyn.cm^−2^ compared to static condition, respectively (Additional file 2: Table S3a). HMOX1 is a NRF2-sensitive gene containing an ARE-sequence and exerts important neuroprotective effects. Several publications already reported the upregulation of its mRNA and protein levels by the flow [61, 62]. HMOX1 metabolizes heme to produce carbon monoxide, bilirubin, and iron which has strong antioxidant and antiviral functions. At the BBB level, carbon monoxide production reduces the release of neuroinflammatory mediators, including interleukin-1β (IL-1β). This cytokine is actually predicted to be significantly inhibited in both fluidic conditions, as well as a high number of other inflammatory cytokines, including transforming growth factor-β (TGF-β), tumor necrosis factor (TNF), and interleukins (Additional file 2: Table S5). Besides the anti-inflammatory action of HMOX1, NRF2 also attenuates the inflammatory effects induced by ROS generation. NRF2 is known to decrease expression of Stimulator of Interferon Genes (STING), thus repressing the transcription of inflammatory genes, as well as production of type I interferons [63]. In this work, interferons pathways were significantly predicted to be inhibited, but also their causal network, with activation z-scores < -2.9 (Additional file 2: Table S5). Homeostasis requires a tight regulation of interferon production. A balanced synthesis of type I interferons is necessary to protect the cells against viral infection. Indeed, an excessive production leads to a disproportionate immune response correlated with the development of several pathologies. Thus, down-regulation of interferon synthesis could be associated with decreased inflammation. A graphical representation of the differential expression of proteins involved in interferon signaling in antiviral response is provided in Fig. 7.

Furthermore, our list of inhibited proteins (Additional file 2: Table S3b) revealed several proteins implicated in immune responses. This includes retinoic acid-inducible gene I receptor (DDX58), an important RNA sensor in the viral recognition, but also interferon-induced GTP-binding protein Mx2, interferon-induced protein 44 (IFI44), and protein involved in leukocyte adhesion to EC, like intercellular adhesion molecule 1 (ICAM1) and connective tissue growth factor (CTGF). Signal transducer and activator of transcription 1 (STAT1) also belongs to the list of proteins significantly inhibited by SS, and reduction of its expression represses the transcription of its target genes that stimulates inflammation and inhibits cellular differentiation. Annexin A1 is significantly over-expressed by SS, with fold changes of 1.2 and 1.5 at 5 and 10 dyn.cm^−2^ compared to static culture, respectively (Additional file 2: Table S3a). This anti-inflammatory protein was previously documented regarding its protective effect on BBB integrity [64, 65].

Some studies reported the reduction of adhesion molecules expression in sheared EC, as ICAM-1 and CTGF [9, 66]. In vivo, atheroprotected sites of the endothelium also exhibit lower expressions of *adherens* junction proteins. Otherwise, some interferon regulatory factors (IRF7, -3, and -1), which translocate to the nucleus to induce transcription of type I interferon, were predicted inhibited by IPA (Additional file 2: Table S5). Other pathogen recognition receptors (Toll-like receptors TLR7 and TLR9) were identified by IPA as inhibited upstream regulators as well as TNF, STAT2, and STAT3.

By contrast, we can notice that Krüppel-like factor 2 (KLF2) is suggested to be an induced upstream regulator (activation z-scores of 1.94 in the two fluidic conditions, Additional file 2: Table S5). KLF2 promoter was elsewhere reported to be induced after a prolonged laminar flow in EC, thus elevating its mRNA and protein levels both in vitro and in vivo [67–69]. This key transcriptional factor was previously suggested to be involved in the activation of up to one third of all flow-responsive genes [70] and to be beneficial in vascular development and in homeostasis and cell quiescence. Among its anti-inflammatory effects, the suppression of the pro-inflammatory activation of monocytes [71], and repression of IL-8 and IL-6 cytokines [72] were reported. KLF2 was also shown to participate to the induction of NRF2 activity by decreasing caveolin-1 expression [72] Caveolin-1 is a mechano-sensor significantly down-regulated in hCMEC/D3 cells, with fold changes of – 1.7 and – 2.3 after respective exposure to 5 and 10 dyn.cm^−2^, together with the associated proteins caveolae-associated protein 1 and 2 (PTRF and SDPR) (with fold changes going from – 1.7 to – 3.3) (Additional file 2: Table S3b). In EC, KLF2 can down-regulate NF-κB, an upstream regulator which appeared significantly inhibited in the two SS conditions (activation z-scores < – 2, Additional file 2: Table S5). NF-κB is another flow-responsive nuclear factor reported to be induced within the first hours of laminar physiological flow, but repressed in case of prolonged SS, consistently with our observation [73]. It was noticed that physiological SS could affect the NF-κB functions by restricting its ability to induce inflammatory gene expression, and by promoting the transcription of cytoprotective NF-κB-dependent molecules [74]. Down-regulation of this pro-inflammatory nuclear factor can be linked to the inhibition of leukocyte binding to EC but also cell cycle (functions which present significant activation z-scores < – 2, Fig. 3a). In vivo, NF-κB was noted to contribute to the protection against atherosclerosis. Conversely, regions where SS is low or disturbed showed an activation of NF-κB, which leads to a local inflammation and the recruitment of monocytes [74, 75]. In order to promote proliferation and inflammation, NF-κB notably induces hypoxia-inducible factor 1-alpha (HIF-1α) transcription [75]. In consistence with reduced expression of NF-κB, we found here that both HIF-1α and its causal networks present high rates of inhibition according to the IPA predictions, with significant activation z-scores < – 2 after fluidic exposure (Additional file 2: Table S5). In addition, the high level of HMOX1 observed in this study is also consistent with negative regulation of NF-κB and HIF-1α, since HMOX1 is negatively regulated by HIF-1α in the brain [76, 77]. HIF-1α can also affect critical vascular functions including angiogenesis, metabolism, and cell growth.

Taken together, our results indicate that exposure of hCMEC/D3 cells to a laminar physiological SS is accompanied by a reduced inflammation, supported by the enhancement of cytoprotective proteins and the simultaneous negative regulation of pro-inflammatory proteins, which correlates with in vivo observations [78]. This reduced inflammation is probably mediated by KLF2, NF-κB, and HIF-1α transcription factors among others.

### Shear stress inhibited cytoskeleton-associated functions

Cytoskeleton is implicated in a multitude of cellular functions, including the response to laminar flow. We found here that cytoskeleton-associated functions, such as actin cytoskeleton signaling, microtubule dynamics, and organization of cytoskeleton, had negative z-scores, suggesting a possible inhibition by SS (Fig. 3). In particular, eight proteins involved in actin filament-based process were down-regulated by SS with high significance; those include band 4.1-like proteins-2 and -3 (EPB41L2 and EPB41L3), pro-apoptotic WT1 regulator (PAWR), myristoylated alanine rich protein kinase C substrate (MARCKS), SUN domain-containing protein 2 (SUN2), tropomyosin alpha-1 chain (TPM1), myosin-9 and -10 (MYH9 and MYH10) (Additional file 2: Table S3b). Conversely, some cytoskeleton-related proteins were overexpressed by SS, including SLC9A3 regulator 2 (SLC9A3R2), gelsolin (GSN), inverted formin 2 (INF2), and fascin actin-bundling protein 1 (FSCN1) (Additional file 2: Table S3a). SLC9A3R2 acts as a scaffold by connecting the membrane proteins to the actin cytoskeleton and regulates their expression; GSN regulates the morphology of the stress fibers. INF2 cuts the actin filaments to accelerate its remodeling; while FSCN1 is an actin bundling protein regulating the cytoskeletal structures. Moreover, we can note that nesprin-3 (SYNE3) was only quantified in the fluidic proteomes, and was therefore induced by SS. This protein plays a role in the transmission of mechanical forces into the nucleus, and regulates the cell morphology [79].

**Fig. 8:**
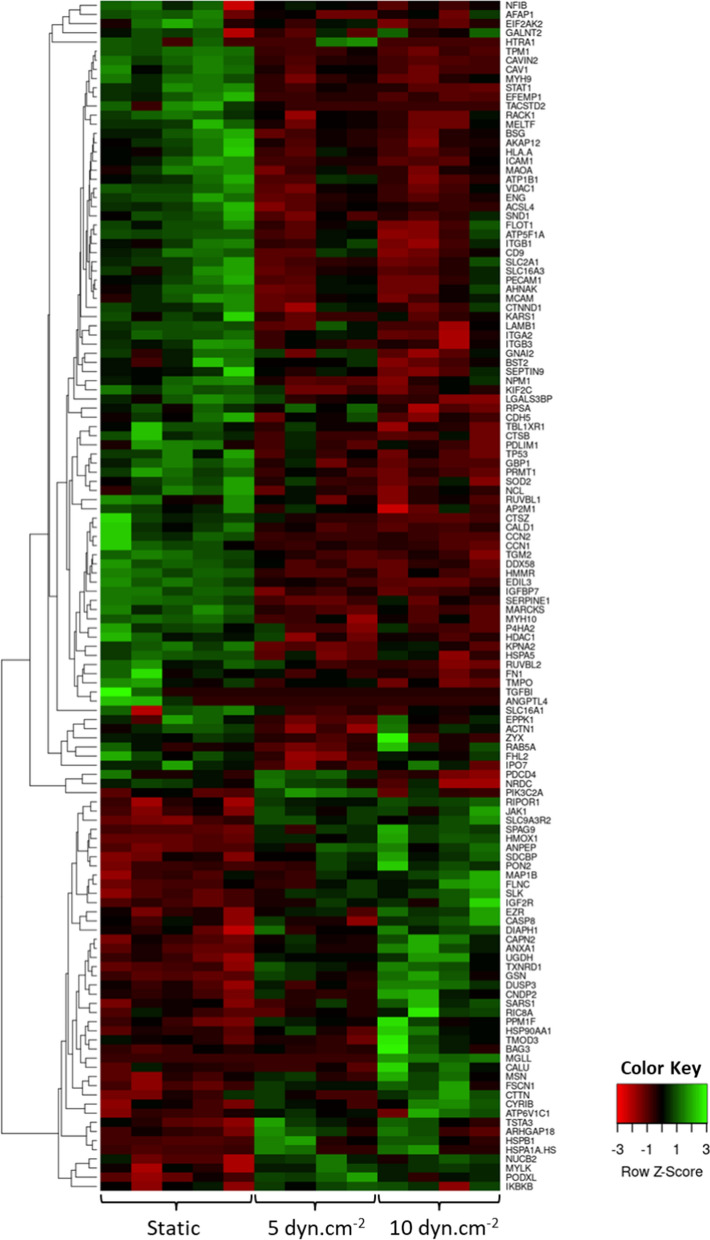
Expression profile of proteins involved in the pathway “migration of cells” for each replicate in the different culture conditions (static culture, 5 or 10 dyn.cm^−2^ shear stress). This heatmap was created with Heatmapper

Among regulated functions, RhoA signaling had negative activation z-scores after SS exposure (Fig. 3). This small GTPase previously demonstrated key roles in the dynamic organization of the actin cytoskeleton [80], but also in associated cellular functions notably cell morphology, adhesion, motility, cytokinesis, cell cycle regulation, and proliferation [81]. Interestingly, all these functions (cell migration, cell movement, adhesion of vascular EC, M phase, and cytokinesis) were predicted to be decreased by SS with a significant activation z-scores < –2 in the two SS conditions (Fig. 3). Both RhoA signalling and their downstream functions were inhibited after 72 h of flow. It was shown that SS transiently increased RhoA activity as early as the first hour of exposure to initiate the cell contraction and formation of stress fibers, but then led to its down-regulation [82]. RhoA inhibition was already reported in bovine primary BMEC cultured one day at 10 dyn.cm^−2^ [80]. More recently, DeOro et al. reported in a pre-print an inhibition of RhoA in hCMEC/D3 cells (4 days at 2.1 dyn.cm^−2^) [83], consistent with our results at both 5 and 10 dyn.cm.^−2^. Functions related to the motility were also significantly inhibited by SS at 72 h (Fig. 3). Of note, cell migration was a function significantly predicted to be decreased, with activation z-scores < –2 after SS exposure (Fig. 3a). All the proteins involved in the regulation of these functions are presented in Fig. 8. These observations are in line with results in EC, showing that cytoskeleton remodelling induced by SS is accompanied by a reduction of EC motility [84]

Inhibition of RhoA activity (as well as NF-κB, as cited above) can also be associated with changes in cell cycle and proliferation. Here, the mitotic phase was significantly inhibited by SS, indicating that the cell division process was slowed down. On the other hand, the Ki-67 proliferation marker protein was down-regulated in both fluidic cultures with fold changes of -1.4 and p-values < 0.01 (Additional file 2: Table S3b). Cell viability was also significantly modulated by SS, with activation z-scores of – 0.66 and – 0.98 at 5 and 10 dyn.cm^−2^ versus static, respectively (Fig. 3a). Apoptosis and necrosis seem affected by SS due to significant activation z-scores, even if the prediction was unclear (Fig. 3a). Inhibition of the cell cycle was well described in response to physiological laminar SS in EC. In vivo, EC exposed to laminar SS exhibited low proliferation levels, in contrast to EC in flow disturbed-regions [85]. This observation was confirmed in vitro since BMEC cultured under laminar physiological SS were stopped in G0 or G1 phase of the cell cycle [86]. The reduction of cell proliferation evoked by SS was reported to favor endothelial integrity [87]. Taken together, these observations indicate that cell motility and turnover were inhibited in hCMEC/D3 cells after long-term exposure to SS. These properties are consistent with the SS-induced physiological quiescent state of BMEC [88].

### Shear stress induced reorganisation of junctional proteins

**Fig. 9:**
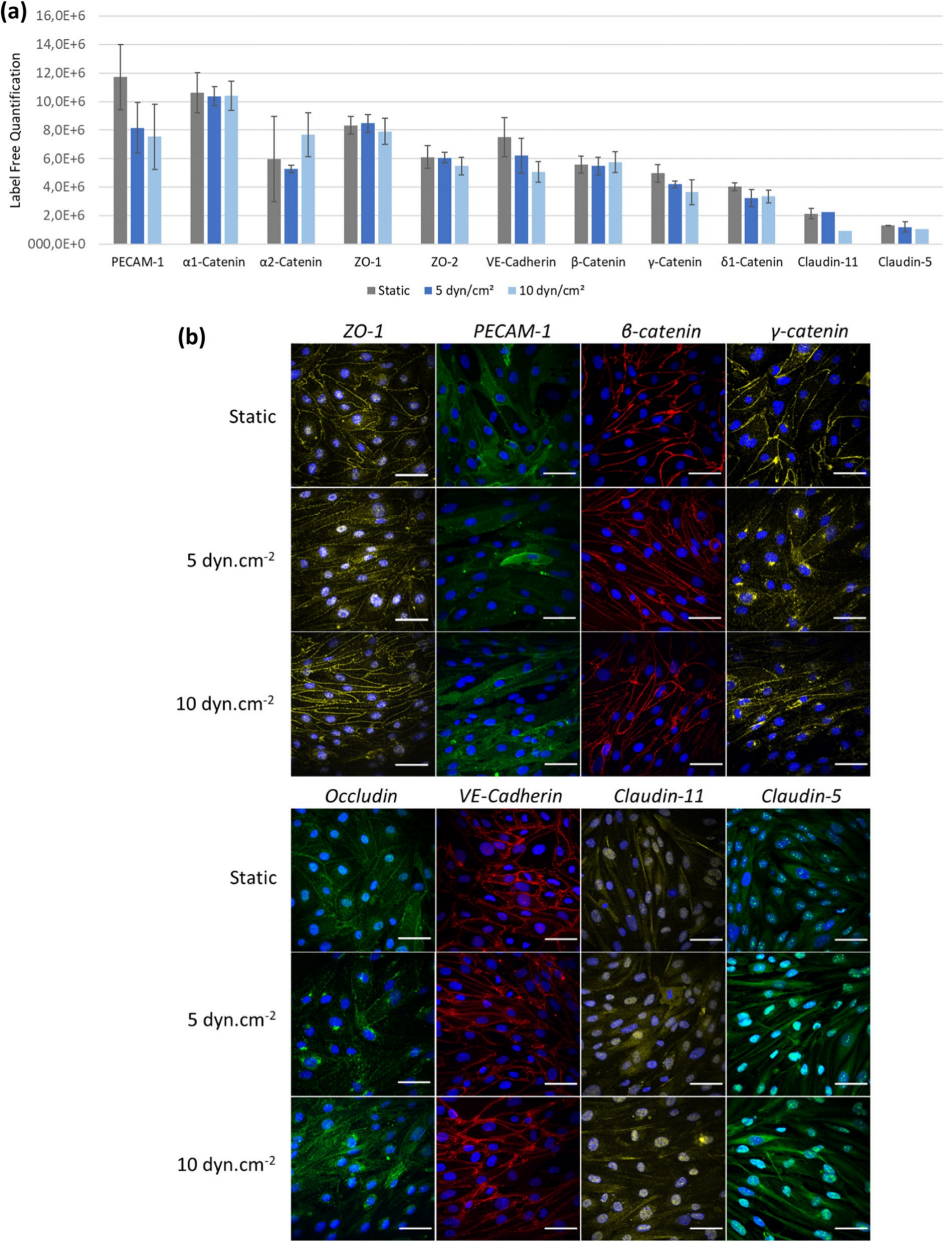
Impact of shear stress exposure on junctional BBB proteins. **a** Label Free Quantification (n = 4 to 5) and (**b**) immunofluorescence staining of indicated junctional proteins determined in hCMEC/D3 cells cultured in static condition or under shear stress of 5 dyn.cm^−2^ or 10 dyn.cm^−2^ during 4 days after proliferation phase. Each image is representative of four images taken in random locations (nucleus in blue, scale = 50 µm)

Inter-endothelial junctions constitute a class of important proteins, which are key elements of the BBB properties and tightness. Among tight junction proteins, Claudin-5 and Claudin-11 were quantified in both static and fluidic culture conditions, as well as the associated cytoplasmic proteins zonula occludens-1 and -2 (ZO-1 and ZO-2). Regarding *adherens* junctions, α1- α2-, β-, δ1-, γ-catenin, VE-cadherin and PECAM-1 (platelet/endothelial cell adhesion molecule-1) were detected. Claudin-5 was the lowest expressed junctional protein. These observations are consistent with previous transcriptomic data reporting a low expression of Occludin and Claudin-5 in hCMEC/D3 cells as compared to primary BMEC [89]. Results presented in Fig. 9a show that SS exposure did not significantly affect the expression of any of these junctional proteins. Targeted proteomic experiments in hCMEC/D3 cells (cultured without SS or under 5 dyn.cm^−2^) confirmed these data, since no significant differences were noted in both claudin-5 and PECAM-1 expressions. Expression ratio were 1.07 $\pm$0.44 and 0.94 $\pm$ 0.10 for claudin-5 and PECAM-1 in dynamic culture compared to the static condition, respectively.

To go further, organization and cellular localization of these key BBB junctional proteins were investigated by immunofluorescence analysis (Fig. 9b). While β-catenin and VE-cadherin were exclusively expressed at the cell–cell contacts, localization of other junctional proteins was more diffuse. PECAM-1 was mostly cytoplasmic, and only a proportion of the stained cells expressed this protein, regardless of the culture conditions. Claudin-5 and -11 were more expressed in the cytoplasm and as bundles in the nucleus than at the intercellular space and SS exposure seemed to increase Claudin-5 expression at 10 dyn.cm^−2^. ZO-1 intensities decreased after exposure to 5 dyn.cm^−2^, but well increased at the cell membrane level at 10 dyn.cm^−2^. Occludin expression was reduced in the whole cellular space (nucleus, cytoplasm, and membrane) at 5 dyn.cm^−2^, and appeared more likely cytoplasmic at 10 dyn.cm^−2^. These immunostainings revealed some changes in both fluorescence intensity and localization of ZO-1, γ-catenin, Occludin, and Claudin-5 between static and fluidic conditions, suggesting a possible reorganization of these junctions under flow.

To our knowledge, only three publications reported a strict comparison of junctional protein expressions between hCMEC/D3 cells cultured under SS and static condition. Brown et al. showed that ZO-1 and Claudin-5 intensities at the cell membrane were increased after 2.73 dyn.cm^−2^ exposure for 18 h in a parallel-plate flow dual chamber [45]. DeOre et al. confirmed the increase in ZO-1 expression by immunofluorescence [83]. They cultivated hCMEC/D3 cells with astrocytes in artificial capillaries with SS from 0.18 to 2.1 dyn.cm^−2^ for 4 days. Finally, Walter et al. used hCMEC/D3 in co-culture with both astrocytes and pericytes in a parallel-plate flow dual chamber [47]. Any difference was noted after exposure to 0.15 dyn.cm^−2^ SS for 2 days. Otherwise, a recent gene expression profiling analysis revealed that the transcript level of some claudins decreased with SS, but the transcripts of other junctions cited above remained unchanged [90]. These studies highlighted strong variations in experimental conditions, but also in responses to SS. In these studies, hCMEC/D3 cells were seeded at the interface between two compartments. In contrast, in our work, cells were cultivated at the bottom of a single channel. In the future, this experiment could be reproduced after cultivating hCMEC/D3 cells on a dual chamber microfluidic device composed of a semi-permeable membrane [91]. Effects of SS on BMEC of various origins was previously reported: de Stefano et al. reported no clear differences between claudin-5, occludin, and ZO-1 expression/localization under static and flow conditions in derived human brain microvascular endothelial cells (dhBMECs) [50], whereas SS triggered the translocation of ZO-1 with an increased junctional intensity in primary porcine BMEC[92] and a stronger immunoreactivity of occludin and ZO-1 at cell borders in primary bovine BMEC[93].

#### Shear stress regulated expression of some blood–brain barrier transporters

**Fig. 10:**
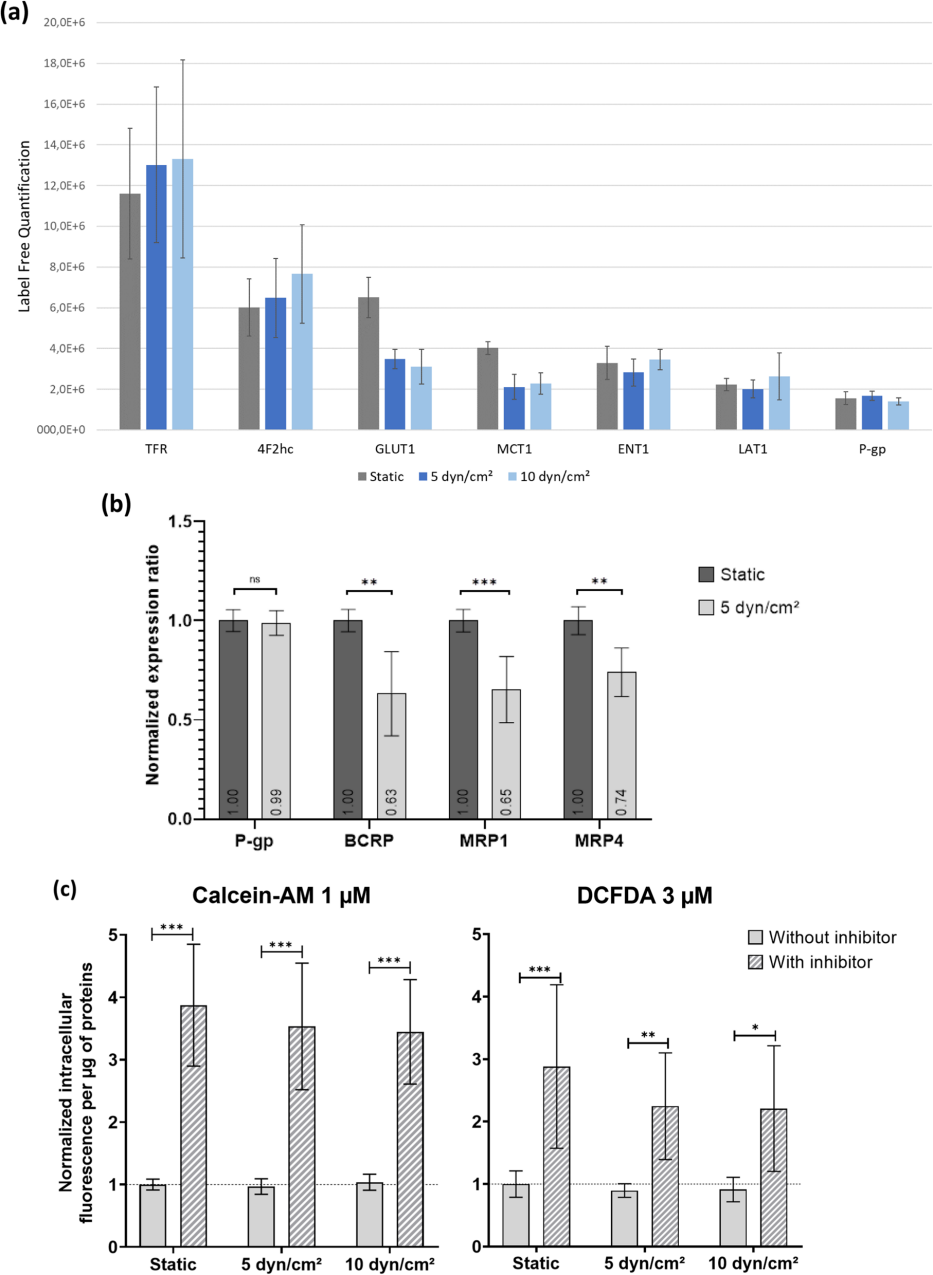
Protein levels of transporters and receptor in hCMEC/D3 cells cultured either in static or under shear stress exposure of 5 or 10 dyn.cm^−2^. Data are presented as means $\pm$standard deviation (**a**) Label free quantification (n = 4 to 5) (**b**) Targeted proteomic analysis of ABC transporters from two independent experiments in triplicates. Expression ratio are normalized with the corresponding value in static. **c** Functionality of P-gp and MRP transporters. Calcein-AM or DCFDA were respectively incubated for 30 min with or without specific inhibitor (respectively Valspodar or MK-571). Histograms present the cellular uptake of fluorescent metabolites of substrates normalized with the value static condition without inhibitor. Three independent experiments were performed in triplicate. Statistical significance was determined by ANOVA followed by a Tukey’s test (*p < 0.05; **p < 0.01; ***p < 0.001)

Finally, we further focused on the effect of SS on some BBB receptors and transporters. The whole proteomic analysis revealed the expression profile of receptors and transporters in hCMEC/D3 cells under both culture in static or SS conditions (Fig. 10a). Among transcytosis receptors, transferrin receptor 1 (TfR1) was detected at high levels, while the insulin receptor (INSR) and low-density lipoprotein receptor‐related protein 1 (LRP1) were not quantified. These relative protein abundances were in accordance with previous proteomic studies on hCMEC/D3 cells [28], isolated human brain microvessels [94], and human primary BMEC [89], showing the decrease in INSR and LRP1 expression in in vitro BBB models compared to in vivo materials [89]. Regarding solute carrier (SLC) transporters, the glucose transporter (GLUT1) was the most expressed protein, followed by 4F2hc, the monocarboxylate transporter 1 (MCT1), the equilibrative nucleoside transporter 1 (ENT1) and the large neutral amino acid transporter (LAT1). The proportion of these SLC transporters revealed by whole proteomics analysis was consistent with that observed in human brain microvessels [94], but differed in another study [28]. Other drug transporters were not detected, such as di-peptide transporters (PEPTs), organic anion transporters (OATs), organic anion‐transporting polypeptides (OATPs), organic cation transporters (OCTs), organic cation/carnitine transporters (OCTNs) and multidrug and toxic compound extrusions (MATEs), these proteins being under the limit of quantification in some proteomic studies [28, 94]. Regarding ATP-binding cassette (ABC) efflux transporters, P-glycoprotein (P-gp) was the only ABC efflux transporter detected by this global proteomic analysis while Breast cancer resistant protein (BCRP) and multidrug resistance-associated proteins (MRPs) were not detected (Fig. 10a).

We thus performed a targeted proteomic analysis to quantify their absolute amounts. This analytically validated method [27] is very specific and makes it possible to detect and quantify proteins (via their proteotypic peptide) even when weakly expressed, unlike the non-targeted approach. Indeed, the quantification of expression of BCRP, MRP1 and MRP4 (Fig. 10a) was possible in the targeted approach (Fig. 10b). The normalized protein expression levels of these transport proteins were presented in Fig. 10b for hCMEC/D3 cells cultured in static and under SS at 5 dyn.cm^−2^. Firstly, this experiment confirmed that P-gp expression levels were not affected by SS and that P-gp was expressed at higher levels than the other ABC efflux pumps. This observation was consistent with previous studies on hCMEC/D3 cells [28, 95–97], although in isolated brain microvessels, BCRP was the most abundant ABC transporter [94]. Our results also showed that SS induced a significant reduction of BCRP protein expression levels, that decreased at 63.2% $\pm$21.2 as compared to static condition. Protein expression levels of MRP1 and MRP4 were also significantly decreased under SS (5 dyn.cm^−2^), with percentages of 65.3 $\pm$ 16.7 and 74.0 $\pm$ 12.2 relative to the static culture, respectively. It is interesting to note that MRP1 is not expressed in normal human BMEC, but induced in pathologic contexts [98]. Our results show that ABC transporters undergo different responses to flow. In the literature, the transcriptional expression of *ABCB1* (encoding for P-gp), *ABCC1* (MRP1), *-2* (MRP2) and *-5* (MRP5), but not *ABCC3* (MRP3), was reported to be induced by SS in primary human BMEC [17] and in the HBMEC cell line [99]. In contrast, DeStefano et al. reported no change in *ABCB1* transcript levels in BMEC-like cells derived from human pluripotent stem cells [50].

In order to assess whether the differences in expression levels of MRPs could affect their efflux capacities, MRPs functional activity was quantified. P-gp activities were also analyzed for comparison (Fig. 10c). Significant efflux activities were observed for each condition, providing evidence of P-gp and MRPs functional activities in hCMEC/D3 cells. However, there were no significant differences between the culture conditions. The absence of variations in both P-gp expression levels and activity was thus confirmed with functional activity experiment. SS exposure showed no significant impact on MRPs activities in control conditions (without inhibitors), even if the difference of MRP activity with and without MRPs inhibitor decreased with SS magnitudes.

## Conclusion

Shear stress provides multifaceted effects on the proteome of the human cerebral microvascular hCMEC/D3 cells. We confirmed that these brain microvascular ECs are able to detect and respond to SS, by intracellularly transmitting this hemodynamic force. hCMEC/D3 cells respond to SS exposure at both 5 and 10 dyn.cm^−2^, notably by elongating and aligning perpendicularly to the flow. This phenomenon could be interpreted as a specific response to flow in BMEC. It would be thus important to repeat these experiments with other human brain endothelial cells like primary BMEC or iPSC-derived cells to validate our results with hCMEC/D3 cells. Another potential limitation of this work was the use of soluble factors like bFGF in the culture medium of hCMEC/D3 cells which can modify responses to SS. Moreover, results provided in this work indicated that hCMEC/D3 cells adapted their protein expression levels according to the magnitude of SS. The principal responses to SS identified here were activation of antioxidant and anti-inflammatory pathways, as well as reduction of cell motility and turnover. They strongly indicate an endothelial protective effect of SS, suggesting a vascular protective action at the BBB. Our results provide elements to compare the responses to flow of BMEC and peripheral ECs, although the underlying mechanisms of these pathways still require further investigations, particularly regarding the BBB phenotype. Finally, this work not only demonstrates the usefulness of SS for improving hCMEC/D3-based in vitro BBB models, but strongly suggests that shear stress may impact on the phenotype of brain endothelial cells in vivo; further experiments would be required in particular to explore whether variation in SS intensity could be responsible for differences in gene/protein expression in BMEC from various brain vessels, namely arterioles, venules and capillaries.

## Supplementary Information

**Additional file 1: **Full crude dataset of proteomics study.

**Additional file 2: Table S1.** Target peptides and selected ions used in the LC-MS/MS multiplexed MRM method analysis, according to the protocol previously reported [95]. **Table S2.** Characteristics of primary antibodies used for immunostaining. **Table S3.** Significantly (**a**) upregulated and (**b**) downregulated proteins in hCMEC/D3 cells cultured under shear stress (both 5 and 10 dyn.cm^-^²) for 72 hours compared to the static culture. Selection criteria were |fold change| > 1.2 and both ANOVA and Student t-test p-values < 0.01, plus ON and OFF proteins (respectively annotated + and –).**Table S4.** Significantly upregulated and downregulated proteins in hCMEC/D3 cells cultured under shear stress (at 5 or 10 dyn.cm^-^²) for 72 hours compared to the static culture . Selection criteria were |fold change| > 1.2 and both ANOVA and Student t-test p-values < 0.05. **Table S5.** Upstream regulators identified by Ingenuity Pathway Analysis (Qiagen), and their target molecules. Comparison was made between the proteomes at 5 or 10 dyn.cm^-2^ shear stress versus static culture) appeared to be upstream regulators according to Ingenuity Pathway Analysis (Qiagen). **Figure. S1**. Orientation of hCMEC/D3 cells cultured in static condition (blue) or exposed to an SS of 5 dyn/cm² (orange) or 10 dyn/cm² (red), in the Ibidi µ-Slides (solid line). The graph reports the calculations obtained using the OrientationJ plug-in of the FIJI software, as means of the triplicates with or without standard deviation. The 0° angle corresponds to the axis of the channel (as well as the direction of the flow), and the angles – 90° and 90° correspond to the orientation perpendicular to the channel

## Data Availability

The mass spectrometry proteomics data have been deposited to the ProteomeXchange Consortium via the PRIDE [1] partner repository with the dataset identifier PXD030493. Datasets are available at: Project Name: Proteomic profiling of hCMEC/D3 cells exposed 3 days to laminar physiological shear stress. Project accession: PXD030493. Project DOI: Not applicable. Reviewer account details: Username: reviewer_pxd030493@ebi.ac.uk. Password: 2ejuEa5r. [1] Perez-Riverol Y, Csordas A, Bai J, Bernal-Llinares M, Hewapathirana S, Kundu DJ, Inuganti A, Griss J, Mayer G, Eisenacher M, Pérez E, Uszkoreit J, Pfeuffer J, Sachsenberg T, Yilmaz S, Tiwary S, Cox J, Audain E, Walzer M, Jarnuczak AF, Ternent T, Brazma A, Vizcaíno JA (2019). The PRIDE database and related tools and resources in 2019: improving support for quantification data. Nucleic Acids Res 47(D1):D442-D450 (PubMed ID: 30,395,289).
