# Supplementary material for: Exposure of human cerebral microvascular endothelial cells hCMEC/D3 to laminar shear stress induces vascular protective responses
Source: Fluids Barriers CNS. 2022 Jun 3;19:41. doi: 10.1186/s12987-022-00344-w (PMC9164338; doi:10.1186/s12987-022-00344-w)
Supplement: Supplementary file 2 — Additional file 2: Table S1. Target peptides and selected ions used in the LC-MS/MS multiplexed MRM method analysis, according to the protocol previously reported [95]. Table S2. Characteristics of primary antibodies used for immunostaining. Table S3. Significantly (a) upregulated and (b) downregulated proteins in hCMEC/D3 cells cultured under shear stress (both 5 and 10 dyn.cm-²) for 72 hours compared to the static culture. Selection criteria were |fold change| > 1.2 and both ANOVA and Student t-test p-values < 0.01, plus ON and OFF proteins (respectively annotated + and –).Table S4. Significantly upregulated and downregulated proteins in hCMEC/D3 cells cultured under shear stress (at 5 or 10 dyn.cm-²) for 72 hours compared to the static culture . Selection criteria were |fold change| > 1.2 and both ANOVA and Student t-test p-values < 0.05. Table S5. Upstream regulators identified by Ingenuity Pathway Analysis (Qiagen), and their target molecules. Comparison was made between the proteomes at 5 or 10 dyn.cm-2 shear stress versus static culture) appeared to be upstream regulators according to Ingenuity Pathway Analysis (Qiagen). Figure. S1. Orientation of hCMEC/D3 cells cultured in static condition (blue) or exposed to an SS of 5 dyn/cm² (orange) or 10 dyn/cm² (red), in the Ibidi µ-Slides (solid line). The graph reports the calculations obtained using the OrientationJ plug-in of the FIJI software, as means of the triplicates with or without standard deviation. The 0° angle corresponds to the axis of the channel (as well as the direction of the flow), and the angles – 90° and 90° correspond to the orientation perpendicular to the channel [file 12987_2022_344_MOESM2_ESM.docx]

***Supplemental Table 1.*** *Target peptides and selected ions used in the LC-MS/MS multiplexed MRM method analysis, according to the protocol previously reported* [95]*.*

| **Protein** | **Peptide sequence** | **Isotope type** | **Precursor M/z** | **Product M/z** | **Fragment ion type** |
| --- | --- | --- | --- | --- | --- |
| **P-gp (ABCB1)**  [96] | FYDPLAGK | Light | 455.7 | 763.4 | y7 |
|  |  |  |  | 600.3 | y6 |
|  |  |  |  | 485.3 | y5 |
|  |  | Heavy | 457.7 | 767.4 | y7 |
|  |  |  |  | 604.3 | y6 |
|  |  |  |  | 489.3 | y5 |
| **BCRP (ABCG2)** [95,97] | SSLLDVLAAR | Light | 522.8 | 757.5 | y7 |
|  |  |  |  | 644.4 | y6 |
|  |  |  |  | 529.3 | y5 |
|  |  | Heavy | 524.8 | 761.5 | y7 |
|  |  |  |  | 648.4 | y6 |
|  |  |  |  | 533.4 | y5 |
| **MRP1 (ABCC1)**  [98] | EDTSEQVVPVLVK | Light | 721.9 | 1011 | y9 |
|  |  |  |  | 881.6 | y8 |
|  |  |  |  | 753.5 | y7 |
|  |  |  |  | 555.4 | y5 |
|  |  | Heavy | 725.9 | 1019 | y9 |
|  |  |  |  | 889.6 | y8 |
|  |  |  |  | 761.5 | y7 |
|  |  |  |  | 563.4 | y5 |
| **MRP4 (ABCC4)**  [99] | AEAAALTETAK | Light | 538.3 | 733.4 | y7 |
|  |  |  |  | 662.4 | y6 |
|  |  |  |  | 549.3 | y5 |
|  |  | Heavy | 542.3 | 741.4 | y7 |
|  |  |  |  | 670.4 | y6 |
|  |  |  |  | 557.3 | y5 |
| **PECAM-1**  [95] | NSNDPAVFK | Light | 496.2 | 790.4 | y7 |
|  |  |  |  | 676.4 | y6 |
|  |  |  |  | 561.3 | y5 |
|  |  | Heavy | 500.3 | 798.4 | y7 |
|  |  |  |  | 684.4 | y6 |
|  |  |  |  | 569.4 | y5 |
| **Claudin-5**  [95,97] | EFYDPSVPVSQK | Light | 698.3 | 956.5 | y9 |
|  |  |  |  | 841.5 | y7 |
|  |  |  |  | 558.3 | y5 |
|  |  | Heavy | 702.4 | 964.5 | y9 |
|  |  |  |  | 849.5 | y7 |
|  |  |  |  | 566.3 | y5 |
| **Na^+^/K^+^‐ATPase** [95,97] | AAVPDAVGK | Light | 414.2 | 685.4 | y7 |
|  |  |  |  | 586.3 | y6 |
|  |  |  |  | 489.3 | y5 |
|  |  | Heavy | 417.2 | 691.4 | y7 |
|  |  |  |  | 592.3 | y6 |
|  |  |  |  | 495.3 | y5 |

***Supplemental Table 2.*** *Characteristics of primary antibodies used for immunostaining.*

| **Primary antibodies** | **Reactivity** | **Reference** | **Provider** |
| --- | --- | --- | --- |
| Anti- Claudin-5 | Rabbit | ABT45 | Merck Millipore  (St-Quentin-en-Yvelines, France) |
| Anti- Claudin-11 | Rabbit | 364500 | Invitrogen  (ThermoFisher scientific, Illkirch, France) |
| Anti- ZO-1 | Rabbit | 617300 |  |
| Anti- γ-catenin | Mouse | 138500 |  |
| Anti- β-catenin | Rabbit | ab32572 | Abcam  (Cambridge, UK) |
| Anti- PECAM-1 | Mouse | ab119339 |  |
| Anti- Occludin | Rabbit | ab31721 |  |
| Anti-NRF2  Anti- VE-cadherin | Rabbit  Rabbit | Ab62352  ENZ-ABS661-0100 | Enzo Life Sciences (Villeurbanne, France) |

***Supplemental Table 3.*** *Significantly (****a****) upregulated and (****b****) downregulated proteins in hCMEC/D3 cells cultured under shear stress (both 5 and 10 dyn.cm^-^²) for 72 hours compared to the static culture. Selection criteria were |fold change| > 1.2 and both ANOVA and Student t-test p-values < 0.01, plus ON and OFF proteins (respectively annotated + and –).*

| **(a)** |  |  | |  |  |  |  |
| --- | --- | --- | --- | --- | --- | --- | --- |
| **Up-regulated proteins** | **Gene name** | **5 dyn.cm^-2^** | | **10 dyn.cm^-2^** | |  |  |
|  |  | **Fold change vs static** | **Student**  **p-value** | **Fold change vs static** | **Student**  **p-value** |  |  |
| Nesprin-3 | *SYNE3* | **+** | | **+** | |  |  |
| Isoform 2 of Methionine-R-sulfoxide reductase B3 | *MSRB3* | **+** | | **+** | |  |  |
| Heme oxygenase 1 | *HMOX1* | **10,4** | 7,5E-05 | **16,3** | 2,8E-05 |  |  |
| Fatty acid-binding protein, heart | *FABP3* | **6,3** | 1,8E-04 | **7,8** | 2,4E-03 |  |  |
| NAD(P)H dehydrogenase [quinone] 1 | *NQO1* | **3,9** | 7,1E-05 | **4,1** | 6,1E-05 |  |  |
| Sequestosome-1 | *SQSTM1* | **2,9** | 1,1E-04 | **3,9** | 2,5E-05 |  |  |
| NADP-dependent malic enzyme | *ME1* | **2,8** | 4,2E-03 | **2,7** | 1,8E-04 |  |  |
| Thioredoxin reductase 1, cytoplasmic | *TXNRD1* | **2,2** | 4,4E-03 | **2,5** | 2,0E-03 |  |  |
| Glucose-6-phosphate 1-dehydrogenase | *G6PD* | **2,4** | 2,2E-05 | **2,4** | 2,1E-05 |  |  |
| Glutamate--cysteine ligase regulatory subunit | *GCLM* | **2,4** | 1,4E-04 | **2,3** | 1,9E-04 |  |  |
| Aminopeptidase N | *ANPEP* | **2,0** | 1,6E-03 | **2,2** | 4,0E-04 |  |  |
| Ribosyldihydronicotinamide dehydrogenase [quinone] | *NQO2* | **1,8** | 1,2E-03 | **1,9** | 1,5E-03 |  |  |
| Glutathione reductase | *GSR* | **1,7** | 3,4E-03 | **1,8** | 7,4E-04 |  |  |
| Annexin A4 | *ANXA4* | **1,3** | 2,5E-03 | **1,8** | 4,1E-04 |  |  |
| BTB/POZ domain-containing protein KCTD12 | *KCTD12* | **1,8** | 6,7E-03 | **1,8** | 2,7E-04 |  |  |
| Transketolase | *TKT* | **1,6** | 3,8E-05 | **1,7** | 8,9E-05 |  |  |
| Na(+)/H(+) exchange regulatory cofactor NHE-RF2 | *SLC9A3R2* | **1,7** | 7,9E-04 | **1,7** | 2,3E-03 |  |  |
| SH3 domain-binding glutamic acid-rich-like protein 3 | *SH3BGRL3* | **1,9** | 1,2E-03 | **1,7** | 5,6E-03 |  |  |
| Serine incorporator 1 | *SERINC1* | **2,3** | 2,2E-04 | **1,7** | 9,4E-03 |  |  |
| Transaldolase | *TALDO1* | **1,9** | 3,6E-03 | **1,7** | 2,2E-03 |  |  |
| Glucosamine-6-phosphate isomerase 1 | *GNPDA1* | **1,5** | 9,5E-04 | **1,6** | 3,1E-04 |  |  |
| V-type proton ATPase subunit E 1 | *ATP6V1E1* | **1,5** | 9,6E-04 | **1,6** | 1,8E-03 |  |  |
| Isoform 2 of Gelsolin | *GSN* | **1,3** | 2,2E-03 | **1,6** | 5,6E-04 |  |  |
| Isoform 2 of Tropomyosin alpha-4 chain | *TPM4* | **1,3** | 2,0E-03 | **1,5** | 7,6E-04 |  |  |
| Annexin A1 | *ANXA1* | **1,2** | 6,5E-03 | **1,5** | 4,6E-04 |  |  |
| 6-phosphogluconate dehydrogenase, decarboxylating | *PGD* | **1,5** | 2,1E-03 | **1,5** | 2,8E-03 |  |  |
| Glycogen phosphorylase, brain form | *PYGB* | **1,3** | 3,3E-05 | **1,5** | 2,3E-06 |  |  |
| C-Jun-amino-terminal kinase-interacting protein 4 | *SPAG9* | **1,3** | 4,1E-03 | **1,4** | 8,2E-05 |  |  |
| Delta(3,5)-Delta(2,4)-dienoyl-CoA isomerase | *ECH1* | **1,7** | 5,6E-05 | **1,4** | 7,8E-03 |  |  |
| Ubiquitin-like modifier-activating enzyme 1 | *UBA1* | **1,2** | 9,6E-03 | **1,4** | 4,5E-04 |  |  |
| Cation-independent mannose-6-phosphate receptor | *IGF2R* | **1,2** | 5,8E-03 | **1,4** | 8,1E-03 |  |  |
| Inverted formin-2 | *INF2* | **1,4** | 1,8E-03 | **1,4** | 5,8E-03 |  |  |
| Fascin | *FSCN1* | **1,3** | 1,7E-03 | **1,3** | 3,8E-03 |  |  |
| V-type proton ATPase subunit B, brain isoform | *ATP6V1B2* | **1,2** | 2,6E-04 | **1,3** | 1,9E-04 |  | |
| Isoform 2 of STE20-like serine/threonine-protein kinase | *SLK* | **1.2** | 1,8E-03 | **1.3** | 5,9E-04 |  | |

| **Down-regulated proteins** | **Gene name** | **5 dyn.cm^-2^** | | **10 dyn.cm^-2^** | |
| --- | --- | --- | --- | --- | --- |
|  |  | **Fold change  versus static** | **Student**  **p-value** | **Fold change  versus static** | **Student**  **p-value** |
| Protein scribble homolog | *SCRIB* | **-** | | **-** | |
| von Willebrand factor | *VWF* | **-** | | **-** | |
| NADH dehydrogenase [ubiquinone] 1 beta subcomplex subunit 4 | *NDUFB4* | **-** | | **-** | |
| Probable E3 ubiquitin-protein ligase HERC4 | *HERC4* | **-** | | **-** | |
| Elongator complex protein 3 | *ELP3* | **-** | | **-** | |
| NADH dehydrogenase [ubiquinone] 1 alpha subcomplex subunit 12 | *NDUFA12* | **-** | | **-** | |
| Dihydropyrimidine dehydrogenase [NADP(+)] | *DPYD* | **-** | | **-** | |
| Interferon-induced GTP-binding protein Mx2 | *MX2* | **-3,2** | 2,9E-03 | **-** | |
| NADH-ubiquinone oxidoreductase 75 kDa subunit | *NDUFS1* | **-2,2** | 3,2E-04 | **-7,4** | 9,1E-06 |
| CCN family member 2 | *CTGF* | **-3,8** | 5,6E-05 | **-4,5** | 4,2E-04 |
| CCN family member 1 | *CYR61* | **-2,9** | 1,7E-03 | **-4,3** | 4,1E-03 |
| Interferon-induced protein 44 | *IFI44* | **-2,4** | 3,1E-03 | **-3,9** | 3,9E-03 |
| NADH dehydrogenase [ubiquinone] flavoprotein 1 | *NDUFV1* | **-2,8** | 2,6E-05 | **-3,9** | 1,6E-05 |
| Caveolae-associated protein 2 | *SDPR* | **-2,4** | 2,2E-04 | **-3,3** | 4,3E-06 |
| NADH dehydrogenase [ubiquinone] iron-sulfur protein 2 | *NDUFS2* | **-1,7** | 3,2E-03 | **-2,9** | 4,7E-04 |
| Succinate dehydrogenase [ubiquinone] flavoprotein subunit | *SDHA* | **-2,1** | 1,9E-06 | **-2,8** | 1,3E-06 |
| CDGSH iron-sulfur domain-containing protein 2 | *CISD2* | **-2,0** | 2,7E-04 | **-2,7** | 5,0E-04 |
| NADH dehydrogenase [ubiquinone] 1 alpha subcomplex subunit 9 | *NDUFA9* | **-1,7** | 1,4E-03 | **-2,6** | 2,3E-03 |
| Retinal dehydrogenase 1 | *ALDH1A1* | **-2,8** | 6,0E-03 | **-2,5** | 7,7E-04 |
| UMP-CMP kinase 2 | *CMPK2* | **-1,9** | 3,4E-03 | **-2,4** | 3,9E-03 |
| EGF-like repeat and discoidin I-like domain-containing protein 3 | *EDIL3* | **-1,7** | 2,1E-04 | **-2,3** | 1,1E-05 |
| Kinesin-like protein KIF20A | *KIF20A* | **-2,3** | 4,8E-03 | **-2,3** | 2,3E-03 |
| Palmitoyl-protein thioesterase 1 | *PPT1* | **-1,5** | 3,0E-03 | **-2,3** | 8,7E-04 |
| Endoglin | *ENG* | **-2,3** | 1,0E-03 | **-2,3** | 2,4E-04 |
| Intercellular adhesion molecule 1 | *ICAM1* | **-2,0** | 3,8E-03 | **-2,3** | 2,5E-03 |
| Caveolin-1 | *CAV1* | **-1,7** | 9,5E-03 | **-2,3** | 3,8E-04 |
| Isoform 3 of Tropomyosin alpha-1 chain | *TPM1* | **-1,8** | 2,7E-05 | **-2,2** | 7,3E-06 |
| Hyaluronan mediated motility receptor | *HMMR* | **-2,1** | 2,2E-05 | **-2,2** | 4,0E-04 |
| Protein MGARP | *MGARP* | **-1,8** | 8,4E-03 | **-2,2** | 1,2E-03 |
| Isoform 2 of Procollagen-lysine,2-oxoglutarate 5-dioxygenase 2 | *PLOD2* | **-2,0** | 1,8E-03 | **-2,1** | 1,8E-04 |
| Insulin-like growth factor-binding protein 7 | *IGFBP7* | **-2,1** | 8,5E-04 | **-2,0** | 1,6E-04 |
| Guanine nucleotide-binding protein-like 3 | *GNL3* | **-1,9** | 3,8E-03 | **-2,0** | 6,7E-03 |
| EGF-containing fibulin-like extracellular matrix protein 1 | *EFEMP1* | **-1,7** | 9,1E-04 | **-2,0** | 3,3E-04 |
| Signal transducer and activator of transcription 1-alpha/beta | *STAT1* | **-1,6** | 1,2E-03 | **-1,9** | 3,6E-04 |
| Nuclear factor 1 B-type | *NFIB* | **-1,4** | 3,7E-03 | **-1,9** | 2,8E-03 |
| Protein-glutamine gamma-glutamyltransferase 2 | *TGM2* | **-1,7** | 3,4E-04 | **-1,9** | 8,2E-04 |
| Basigin | *BSG* | **-1,8** | 3,0E-03 | **-1,9** | 6,0E-03 |
| Cytochrome c oxidase subunit 4 isoform 1 | *COX4I1* | **-1,5** | 6,2E-03 | **-1,8** | 2,8E-03 |
| ATP-binding cassette sub-family E member 1 | *ABCE1* | **-1,4** | 1,4E-03 | **-1,8** | 9,9E-05 |
| SUN domain-containing protein 2 | *SUN2* | **-1,9** | 2,8E-04 | **-1,8** | 4,4E-03 |
| Nucleolar complex protein 2 homolog | *NOC2L* | **-1,3** | 1,8E-03 | **-1,8** | 2,7E-03 |
| Inositol-3-phosphate synthase 1 | *ISYNA1* | **-1,4** | 6,2E-03 | **-1,7** | 1,3E-03 |
| NADH dehydrogenase [ubiquinone] 1 alpha subcomplex subunit 10 | *NDUFA10* | **-1,6** | 3,4E-03 | **-1,7** | 6,4E-03 |
| Myristoylated alanine-rich C-kinase substrate | *MARCKS* | **-1,8** | 1,9E-04 | **-1,7** | 1,1E-03 |
| Lysosomal protective protein | *CTSA* | **-1,8** | 6,2E-06 | **-1,7** | 9,7E-04 |
| Eukaryotic translation elongation factor 1 epsilon-1 | *EEF1E1* | **-1,2** | 3,4E-03 | **-1,7** | 1,4E-03 |
| Guanylate-binding protein 1 | *GBP1* | **-1,4** | 9,9E-03 | **-1,7** | 8,2E-05 |
| Caveolae-associated protein 1 | *PTRF* | **-1,7** | 5,2E-03 | **-1,7** | 2,8E-03 |
| EH domain-containing protein 4 | *EHD4* | **-1,4** | 1,9E-04 | **-1,7** | 6,2E-06 |
| Endothelin-converting enzyme 1 | *ECE1* | **-1,6** | 1,4E-03 | **-1,6** | 7,0E-03 |
| Plasminogen activator inhibitor 1 | *SERPINE1* | **-2,0** | 1,8E-05 | **-1,6** | 3,9E-03 |
| Antiviral innate immune response receptor RIG-I | *RIG-I* | **-1,5** | 9,5E-04 | **-1,6** | 1,8E-04 |
| Isoform 2 of Band 4.1-like protein 3 | *EPB41L3* | **-1,6** | 5,2E-04 | **-1,6** | 6,1E-03 |
| Protein FAM107B | *FAM107B* | **-1,6** | 6,9E-03 | **-1,6** | 6,9E-03 |
| DNA topoisomerase 1 | *TOP1* | **-1,3** | 8,0E-03 | **-1,6** | 1,6E-03 |
| ATP synthase subunit d | *ATP5H* | **-1,4** | 8,9E-03 | **-1,6** | 6,4E-04 |
| Voltage-dependent anion-selective channel protein 1 | *VDAC1* | **-1,5** | 2,3E-03 | **-1,5** | 7,9E-04 |
| PRKC apoptosis WT1 regulator protein | *PAWR* | **-1,5** | 2,4E-05 | **-1,5** | 1,5E-03 |
| Stomatin-like protein 2 | *STOML2* | **-1,4** | 6,5E-03 | **-1,5** | 7,4E-03 |
| Long-chain-fatty-acid--CoA ligase 4 | *ACSL4* | **-1,6** | 7,7E-03 | **-1,5** | 1,5E-03 |
| Kinesin-like protein KIF2C | *KIF2C* | **-1,5** | 5,7E-03 | **-1,5** | 2,7E-03 |
| Band 4.1-like protein 2 | *EPB41L2* | **-1,5** | 6,9E-03 | **-1,4** | 3,0E-03 |
| Protein RCC2 | *RCC2* | **-1,3** | 7,0E-04 | **-1,5** | 2,2E-04 |
| Isoform 3 of Protein arginine N-methyltransferase 1 | *PRMT1* | **-1,3** | 1,2E-03 | **-1,4** | 2,0E-04 |
| Proliferation marker protein Ki-67 | *MKI67* | **-1,4** | 4,7E-03 | **-1,4** | 4,8E-03 |
| Septin-11 | *sept-11* | **-1,2** | 9,1E-03 | **-1,4** | 3,5E-04 |
| Protein O-glucosyltransferase 2 | *POGLUT2* | **-1,5** | 3,7E-03 | **-1,4** | 5,6E-03 |
| Isoform 4 of Caldesmon | *CALD1* | **-1,3** | 7,0E-03 | **-1,4** | 3,1E-03 |
| H/ACA ribonucleoprotein complex subunit DKC1 | *DKC1* | **-1,4** | 5,1E-04 | **-1,4** | 4,2E-03 |
| Acetyl-CoA acetyltransferase | *ACAT1* | **-1,4** | 1,1E-04 | **-1,3** | 2,2E-03 |
| Myosin-10 | *MYH10* | **-1,4** | 2,3E-03 | **-1,3** | 5,3E-04 |
| CAD protein | *CAD* | **-1,3** | 5,8E-04 | **-1,3** | 8,0E-03 |
| RNA cytosine C(5)-methyltransferase NSUN2 | *NSUN2* | **-1,3** | 3,7E-06 | **-1,2** | 4,6E-05 |
| Host cell factor 1 | *HCFC1* | **-1,2** | 8,6E-03 | **-1,2** | 3,6E-03 |
| Myosin-9 | *MYH9* | **-1,2** | 1,2E-03 | **-1,2** | 1,9E-03 |

***Supplemental Table 4.*** *Significantly upregulated and downregulated proteins in hCMEC/D3 cells cultured under shear stress (at 5 or 10 dyn.cm^-^²) for 72 hours compared to the static culture . Selection criteria were |fold change| > 1.2 and both ANOVA and Student t-test p-values < 0.05.*

*See Excel file*

***Supplemental Table 5.*** *Upstream regulators identified by Ingenuity Pathway Analysis (Qiagen), and their target molecules. Comparison was made between the proteomes at 5 or 10 dyn.cm^-2^ shear stress versus static culture) appeared to be upstream regulators according to Ingenuity Pathway Analysis (Qiagen).*

***Supplemental Figure 1***


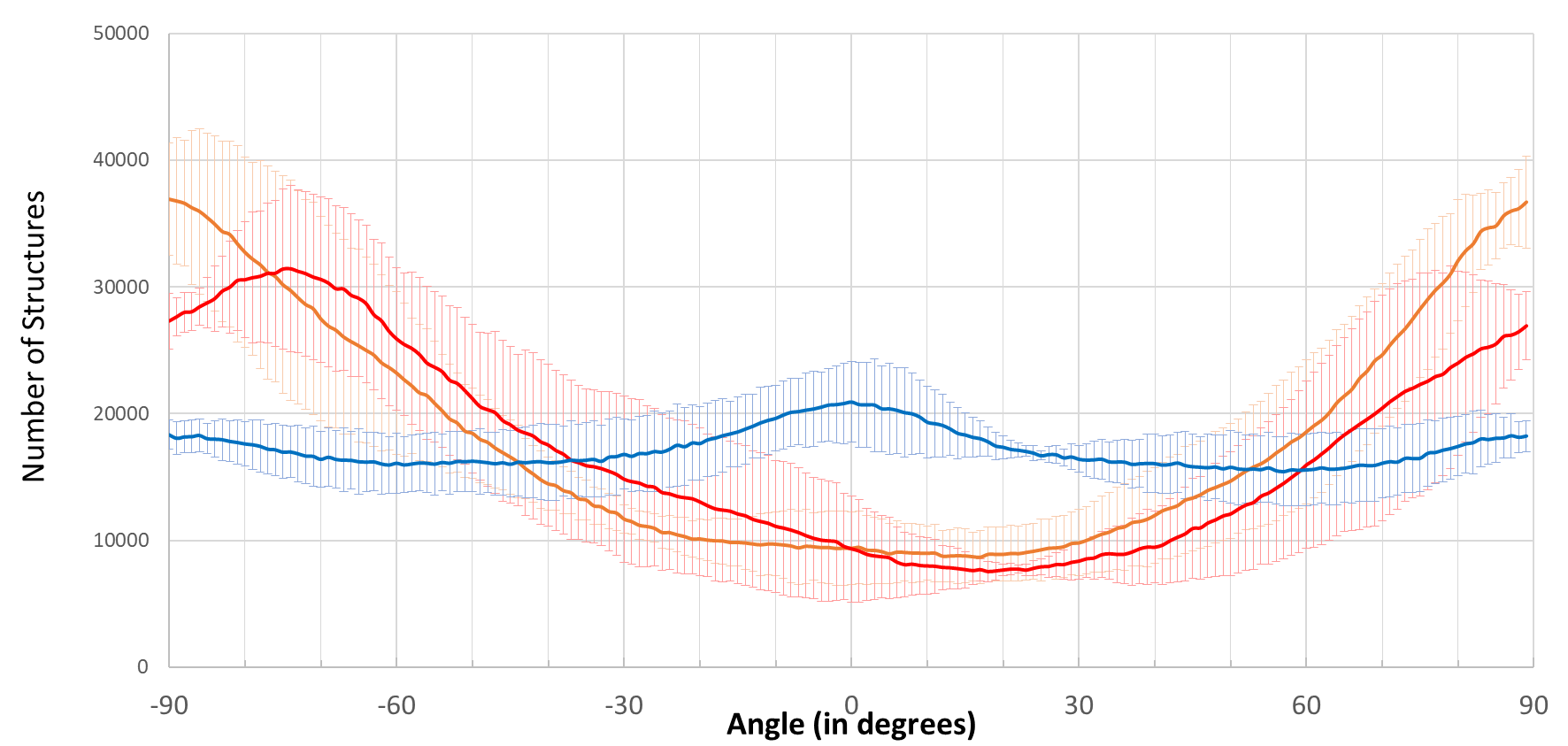


**Supp Fig. 1**. Orientation of hCMEC/D3 cells cultured in static condition (blue) or exposed to an SS of 5 dyn/cm² (orange) or 10 dyn/cm² (red), in the Ibidi µ-Slides (solid line). The graph reports the calculations obtained using the OrientationJ plug-in of the FIJI software, as means of the triplicates with or without standard deviation. The 0° angle corresponds to the axis of the channel (as well as the direction of the flow), and the angles -90° and 90° correspond to the orientation perpendicular to the channel.
